# Photonic Nanomaterials for Wearable Health Solutions

**DOI:** 10.1002/adma.202418705

**Published:** 2025-02-03

**Authors:** Taewoong Park, Jung Woo Leem, Young L. Kim, Chi Hwan Lee

**Affiliations:** ^1^ Weldon School of Biomedical Engineering Purdue University West Lafayette IN 47907 USA; ^2^ Purdue Institute for Cancer Research Regenstrief Center for Healthcare Engineering Purdue Quantum Science and Engineering Institute Purdue University West Lafayette IN 47907 USA; ^3^ School of Mechanical Engineering School of Materials Engineering Elmore Family School of Electrical and Computer Engineering Center for Implantable Devices Purdue University West Lafayette IN 47907 USA

**Keywords:** health monitoring, human skin and eye‐interfaced devices, nanomaterials, photonics, wearable device

## Abstract

This review underscores the transformative potential of photonic nanomaterials in wearable health technologies, driven by increasing demands for personalized health monitoring. Their unique optical and physical properties enable rapid, precise, and sensitive real‐time monitoring, outperforming conventional electrical‐based sensors. Integrated into ultra‐thin, flexible, and stretchable formats, these materials enhance compatibility with the human body, enabling prolonged wear, improved efficiency, and reduced power consumption. A comprehensive exploration is provided of the integration of photonic nanomaterials into wearable devices, addressing material selection, light‐matter interaction principles, and device assembly strategies. The review highlights critical elements such as device form factors, sensing modalities, and power and data communication, with representative examples in skin patches and contact lenses. These devices enable precise monitoring and management of biomarkers of diseases or biological responses. Furthermore, advancements in materials and integration approaches have paved the way for continuum of care systems combining multifunctional sensors with therapeutic drug delivery mechanisms. To overcome existing barriers, this review outlines strategies of material design, device engineering, system integration, and machine learning to inspire innovation and accelerate the adoption of photonic nanomaterials for next‐generation of wearable health, showcasing their versatility and transformative potential for digital health applications.

## Graphical abstract

This review discusses the fundamentals and applications of photonic nanomaterials in wearable health technologies. It covers light‐matter interactions, synthesis, and functionalization strategies, device assembly, and sensing capabilities. Applications include skin patches and contact lenses for diagnostics and therapy. Future perspectives emphasize AI‐assisted design and systematic integration for advancing wearable systems.

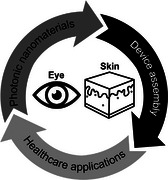

## 1 Introduction

Photonic nanomaterials (NMs) are emerging as indispensable components in wearable health devices due to their unique optical and physical properties.^[^ ^1^ ^]^ Their nanoscale structures enable precise modulation of light absorption, reflection, scattering, and transmission, facilitating accurate detection of key physiological signals such as body temperature, blood glucose, blood pressure, and oxygen saturation (SpO_2_).^[^ ^1^, ^2^ ^]^ These capabilities represent a transformative approach to non‐invasive health monitoring, addressing critical demands for precision and immediate responsiveness in biomedical applications. Compared to conventional electrical‐based wearable sensors, photonic NMs demonstrate distinct advantages, including significantly higher sensitivity, enhanced energy efficiency, and greater durability.^[^ ^3^ ^]^ Electrical sensors often face challenges such as slower response times, limited sensitivity, and vulnerability to electromagnetic interference, which can compromise their reliability in dynamic environments. In contrast, photonic NMs offer rapid, accurate, and interference‐free sensing capabilities, enabling superior performance in wearable applications. Additionally, their ability to leverage light‐based sensing mechanisms for surface‐interaction‐based analyses eliminates the need for invasive procedures such as blood sampling, marking a critical advancement in non‐invasive health monitoring and personalized health. Nanoscale materials such as metal nanoparticles (NPs), quantum dots (QDs), graphene, 2D transition metal dichalcogenides (2D TMDCs), upconversion NPs, and photonic crystals (PCs) are particularly well‐suited for continuous biomedical monitoring, where rapid, reliable, and robust sensing is essential.^[^ ^4^ ^]^ Furthermore, their integration into ultra‐thin, flexible, or stretchable form factors enhances compatibility with the human body, ensuring comfort and adaptability for prolonged wear in daily settings.^[^ ^4^, ^5^, ^6^, ^7^ ^]^

While photonic NMs provide significant advantages, they face limitations compared to electronic devices, which have been refined over decades with optimized manufacturing processes, cost efficiency, and seamless ecosystem integration. Photonic NMs are often associated with higher production costs, complex fabrication methods, and the requirement for specialized optical components, which can complicate their integration into scalable consumer‐grade devices. Additionally, environmental factors such as variable lighting conditions or the opacity of specific biological tissues may affect the performance of photonic NMs, potentially limiting their feasibility in some applications. It is essential to address these limitations for the practical adoption of photonic NMs in health. The multifunctionality of photonic NMs remains a compelling advantage. Their low power consumption supports prolonged operation with minimal energy usage. Also, their capacity to monitor multiple physiological signals simultaneously improves data accuracy and enables comprehensive, data‐driven health management.^[^ ^8^, ^9^ ^]^ By integrating photonic NMs with compact silicon‐based microcircuits, wearable devices can achieve consumer‐grade performance in data processing and wireless communication, fostering seamless interactions among patients, clinicians, and caregivers.

To ensure the successful integration of photonic NMs into wearable health devices, several key requirements must be addressed. These include i) designing ultra‐thin, flexible, or stretchable device form factors for comfortable and secure application to various body areas, ii) developing robust real‐time monitoring capabilities to enable continuous and on‐demand health assessments, and iii) implementing efficient power management and data communication strategies to support compact and user‐friendly systems.^[^ ^4^, ^5^, ^6^, ^10^, ^11^ ^]^ These advancements enable wearable devices to perform body‐interfacing biomedical monitoring while also paving the way for innovative closed‐loop systems that combine multifunctional sensors with therapeutic drug delivery mechanisms.^[^ ^12^ ^]^ By balancing their substantial advantages with a clear understanding of their limitations, photonic NMs have immense potential to revolutionize personalized health and preventive medicine.^[^ ^9^, ^13^ ^]^ Continued research and innovation in this field will drive their adoption, offering transformative solutions that enhance early diagnosis, precise therapy, and continuous health monitoring in modern medical applications.

**Figure 1:**
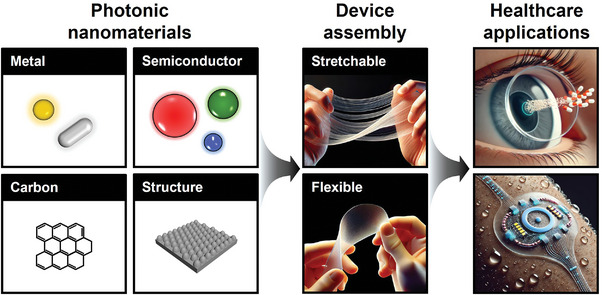
Graphical summary of comprehensive workflow.

In this review, we provide a comprehensive overview of the integration process of photonic NMs into wearable health applications, covering material selection, operational principles, integration strategies, and application methodologies. We highlight the importance of tailoring materials and mechanisms to meet the requirements of specific target applications for skin and eyes (**Figure** **1**). In particular, we focus on designing and assembling precise structures on flexible, biocompatible substrates to achieve optimal functionality. Through examples of health monitoring systems that interface with the skin and eyes, this review demonstrates the seamless integration of photonic NMs into wearable devices. We also detail the construction and implementation methods of these systems, showcasing their potential to advance personalized and preventive health. First, this review begins by providing an overview of photonic NMs, categorizing them based on their chemical bonding and structural characteristics to guide the selection of materials for specific applications. Second, we discuss the principles underlying their light‐matter interaction properties, fabrication engineering, and functionalization, along with key considerations for incorporating photonic NMs into devices. These include aspects such as device form factors, sensing capabilities, and power and data transmission in wearable devices. Third, we examine system‐level applications in health, focusing on diagnostic, therapeutic, and monitoring solutions categorized by skin‐attached and eye‐attached formats. Finally, we address the challenges and obstacles in materials, device design, and system integration, identifying strategies to enable broader adoption of wearable health technologies. This review ultimately aims to inspire future innovations in wearable health devices by addressing both the immense potential and the challenges associated with photonic NMs.

## 2 Introduction to Photonic Nanomaterials

**Figure 2:**
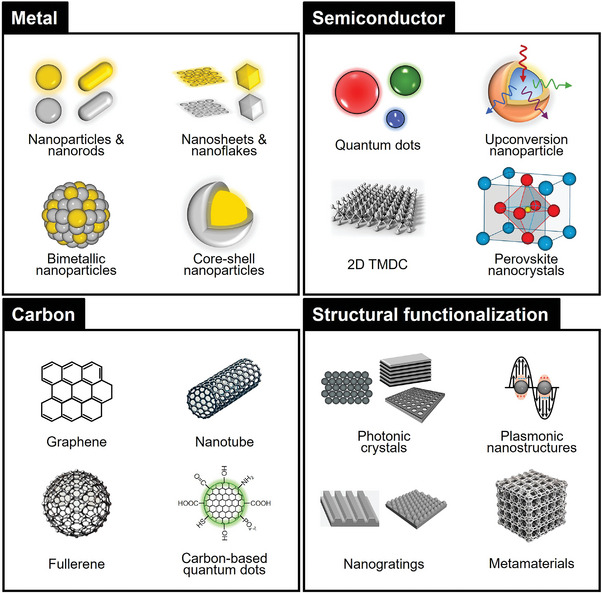
Photonic nanomaterials for wearable health devices.

Photonic NMs are specifically engineered to interact with light at the nanoscale, exhibiting distinctive optical properties determined by their size, shape, and structural composition.^[^ ^14^ ^]^ These materials have catalyzed significant advancements in wearable health technologies, particularly in diagnostics, therapeutics, and monitoring applications. Photonic NMs interact with light in various modes including transmission, reflection, and absorption. Fluorescence and scattering properties can be utilized to modulate signals for detection. The generation of heat or reactive oxygen species (ROS) can be incorporated into therapeutic applications.^[^ ^15^ ^]^ This section categorizes photonic NMs into four main types: i) metal‐based, ii) semiconductor‐based, iii) carbon‐based, and iv) structurally functionalized materials (**Figure** **2**). Each category is explored with a focus on its unique properties, structural characteristics, and specific roles in wearable health devices, highlighting their potential to revolutionize personalized and preventive medicine.^[^ ^2^, ^11^, ^16^ ^]^

### 2.1 Metals

The gold (Au) and silver (Ag) with nanoscale structures are prime examples of metal‐based photonic NMs, offering distinct advantages for diagnostics, therapeutics, and monitoring in wearable health applications. Au NMs, typically sized between 2‒50 nm, are frequently utilized due to their ease of synthesis, stability in aqueous environments, and compatibility with biological functionalization.^[^ ^17^ ^]^ Their localized surface plasmon resonance (LSPR) in the visible spectrum (510–540 nm) enhances biosensing, bioimaging, and drug delivery capabilities.^[^ ^18^, ^19^ ^]^ Functionalization with thiol, phosphine, or amine groups allows conjugation with biomolecules including antibodies and peptides, making Au NMs highly versatile. While concerns exist regarding oxidative stress and genotoxic effects, Au NMs are generally biocompatible and exhibit low cytotoxicity, making them favorable for long‐term biomedical applications.^[^ ^20^, ^21^ ^]^ Ag NMs, typically ranging from 20‒100 nm, exhibit strong plasmon resonance (PR) in the wavelength range of 380‒460 nm, generating intense electromagnetic fields that enhance techniques such as surface‐enhanced Raman scattering (SERS).^[^ ^22^ ^]^ The SERS enables precise biomolecular detection at trace levels.^[^ ^23^ ^]^ Ag NMs also offer cost‐efficiency and intrinsic antibacterial properties,^[^ ^24^ ^]^ which are advantageous for wearable health devices. However, their susceptibility to oxidation can compromise stability in biological environments and lead to cytotoxicity from released Ag ions.^[^ ^25^ ^]^ To solve the limitations, surface modifications using biocompatible coatings such as silica or poly(ethylene glycol) (PEG) are employed, improving oxidation resistance and broadening their applicability in wearable systems.^[^ ^26^ ^]^

The metal‐based NMs can be processed into various nanostructured forms, such as spherical NPs, nanorods, nanosheets, nanoflakes, bimetallic NPs, and core–shell NPs, each with unique properties tailored to specific applications.^[^ ^18^, ^27^ ^]^ Spherical NPs, such as Au and Ag, have been integral to wearable biosensors for their ability to interact with light through LSPR and SERS. Their strong light‐scattering and absorption properties enable precise detection of biomarkers in sweat, tears, or interstitial fluids.^[^ ^28^ ^]^ NPs embedded in skin patches or contact lenses enhance biosensing platforms of glucose levels, dehydration, or inflammation monitoring.^[^ ^2^, ^29^ ^]^ In addition, these NPs can support localized drug delivery in skin patches by releasing therapeutic agents upon light stimulation, targeting skin or eye‐related conditions. Nanorods, with distinct transverse and longitudinal PR modes, are well‐suited for advanced applications. In skin patches, nanorods convert near‐infrared (NIR) light into heat for targeted photothermal therapy (PTT), effectively treating localized infections or cancers.^[^ ^30^, ^31^ ^]^ In contact lenses, facilitate high‐resolution imaging for ocular diagnostics or controlled drug delivery through light‐induced mechanisms.^[^ ^32^ ^]^ Nanosheets and nanoflakes, characterized by their high surface area‐to‐volume ratio, are ideal for flexible, transparent sensors on the skin or eyes.^[^ ^33^ ^]^ They enhance sensitivity to biomolecular changes such as sweat composition or tear biomarkers. Nanosheets integrated into skin patches are used for monitoring hydration levels, pH, or electrolytes while maintaining wearer comfort.^[^ ^34^ ^]^ When used in contact lenses, they are utilized for ocular health monitoring by detecting diseases such as glaucoma or dry eye syndrome.^[^ ^35^ ^]^ Bimetallic and core–shell NPs, such as Au‐Ag or Au‐platinum (Pt) hybrids, combine the properties of multiple metals to enhance optical, catalytic, and antibacterial functionalities.^[^ ^36^ ^]^ In skin patches, Au‐Ag NPs provide infection monitoring through optical sensing while offering antibacterial properties that promote wound healing.^[^ ^37^ ^]^ In contact lenses, Au‐Pt hybrids enhance drug delivery precision and enable dual functionalities for diagnostics and treatment, such as addressing inflammation or bacterial infections.^[^ ^38^ ^]^

### 2.2 Semiconductors

Semiconducting NMs play a pivotal role in wearable health devices due to their high sensitivity to physiological signals, superior electron mobility, and distinctive optical properties. QDs, typically 2–10 nm in size, exhibit strong quantum confinement effects, enabling precise, size‐dependent light emission.^[^ ^39^ ^]^ Cadmium selenide (CdSe) QDs emit light across the visible spectrum (400–700 nm), with smaller dots producing blue‐shifted light due to the increased binding energy of electrons and holes.^[^ ^40^ ^]^ This size‐dependent luminescence makes QDs ideal for multi‐channel sensors that target specific biomarkers, enabling precise monitoring of bio‐signals with high fluorescence efficiency.^[^ ^41^ ^]^ Upconversion nanoparticles (UCNPs), generally 20–30 nm in size, consist of fluoride or oxide matrices doped with lanthanide ions such as ytterbium (Yb^3+^), thulium (Tm^3+^), or erbium (Er^3+^).^[^ ^42^ ^]^ UCNPs absorb NIR light (e.g., 980 nm) and emit visible light (e.g., green at 540 nm or red at 650 nm) through an upconversion process. This property supports non‐invasive, bio‐signal monitoring, such as heart rate and oxygen levels,^[^ ^43^ ^]^ under safe NIR exposure. 2D TMDCs, including molybdenum disulfide (MoS_2_), tungsten disulfide (WS_2_), and bismuth selenide (Bi_2_Se_3_), feature atomic thicknesses (≈0.6–0.7 nm per layer) and consist of transition metals bonded with chalcogens in an MX_2_ configuration.^[^ ^44^ ^]^ The 2D NMs exhibit high electron mobility and strong light absorption across the visible and NIR spectra, making them effective for wearable devices that require precise measurements.^[^ ^45^ ^]^ Perovskite nanocrystals (PNCs), typically with sizes smaller than 20 nm, possess a characteristic ABX_3_ structure (e.g., cesium lead bromide, CsPbBr_3_).^[^ ^46^ ^]^ The PNCs exhibit tunable light absorption and emission, spanning visible to NIR wavelengths.^[^ ^41^, ^47^ ^]^ Black phosphorus (BP), with its layered atomic structure held by van der Waals forces, exhibits strong light absorption across the visible to NIR spectrum and high electron mobility, making it suitable for deeper tissue bio‐signal detection.^[^ ^48^ ^]^ Its flexibility and mechanical resilience enhance its compatibility with skin‐contact devices for long‐term physiological monitoring.

Semiconducting QDs, UCNPs, 2D TMDCs, PNCs, and BP collectively contribute to wearable health devices by leveraging their unique optical properties at the nanoscale for high‐sensitivity biomarker detection. These advancements enhance the accuracy and reliability of wearable devices, supporting surface‐level, sustainable health monitoring systems for long‐term care. Furthermore, the NMs significantly improve the performance of wearable health devices by exploiting their nanoscale properties for precise biomarker detection and monitoring. QDs, with their tunable fluorescence and high quantum yield, enable accurate optical detection of physiological signals such as glucose levels or SpO_2_. UCNPs offer exceptional photostability and deep tissue penetration, making them ideal for long‐term tracking of biomarkers or imaging in low‐light environments. 2D TMDCs provide excellent surface reactivity and electronic properties, facilitating electrochemical sensing of molecules including cortisol or lactate for stress and metabolic monitoring. PNCs combine strong light absorption with high sensitivity, supporting the detection of trace analytes. CsPbBr_3_ PNCs are particularly valuable for oxygen monitoring in skin patches due to their sharp green emission, modulated by oxygen levels. Mixed‐halide perovskites (e.g., CsPbBr_x_I_3‐x_) are employed for multi‐wavelength sensings, such as blood analysis or hydration monitoring via sweat. In contact lenses, methylammonium lead iodide (MAPbI_3_) PNCs are used for monitoring ocular oxygenation and tear composition due to their strong NIR emission. BP, with its unique tunable bandgap and biodegradability, is particularly suitable for transient or bioresorbable devices, offering short‐term monitoring of vital signals without leaving long‐term residues in the body. Together, these NMs enable the development of wearable systems with unparalleled sensitivity, accuracy, and adaptability, supporting non‐invasive and sustainable health monitoring for personalized and long‐term care.

### 2.3 Carbon

Carbon‐based NMs are highly valued for their exceptional electrical, thermal, and mechanical properties, making them integral to wearable health devices. Graphene, a single layer of carbon atoms arranged in a honeycomb lattice, exhibits remarkable electrical conductivity, mechanical strength, and flexibility. With electron mobility reaching up to 2 00 000 cm^2^ (Vs)^‒1^, graphene surpasses many metals, making it ideal for electrodes in biosensors and energy storage systems such as supercapacitors and batteries.^[^ ^49^ ^]^ Its biocompatibility ensures safety in skin‐contact applications, while its optical transparency and mechanical resilience support flexible, bio‐signal monitoring systems.^[^ ^50^ ^]^ Carbon nanotubes (CNTs), formed by rolling graphene sheets into cylindrical structures, exist in single‐walled (SWCNTs) and multi‐walled (MWCNTs) forms. SWCNTs typically have diameters of 1–2 nm, while MWCNTs can reach tens of nanometers. CNTs exhibit extraordinary electrical conductivity and mechanical strength due to their sp^2^ bonding, enabling efficient electron transport. These properties make CNTs highly effective for wearable bio‐signal monitoring sensors, conductive films, and energy storage devices. Additionally, their strong light absorption and emission in the NIR range make them suitable for optical sensors and bioimaging applications.^[^ ^34^, ^51^ ^]^ Fullerenes, such as C_60_, are spherical or symmetrical nanostructures composed of carbon atoms arranged in pentagonal and hexagonal rings. Their sp^2^ bonding imparts stability and electron‐accepting capabilities, making them useful in biosensors and antioxidant therapies. As effective free radical scavengers with biocompatibility, fullerenes find applications in oxidative stress management and drug delivery. Carbon quantum dots (CQDs), typically smaller than 10 nm, exhibit unique photoluminescence (PL) due to quantum confinement effects and mixed sp^2^/sp^3^ bonding. CQDs can absorb light in the UV–vis range (300–500 nm) and emit tunable wavelengths across the visible spectrum based on particle size and surface chemistry.^[^ ^52^ ^]^ CQDs are well‐suited for applications including bioimaging, biomolecule detection, and light‐emitting diode (LED) displays due to their biocompatibility, photostability, and water dispersibility. These properties of CQDs are exploited to detect biological markers with high sensitivity while ensuring non‐toxicity, crucial for safe, long‐term use in wearable devices.^[^ ^53^ ^]^

Graphene, CNTs, fullerenes, and CQDs exhibit unique chemical bonding and nanostructural characteristics that contribute to their high conductivity, mechanical strength, and biocompatibility. Graphene, with exceptional electrical conductivity, supports real‐time signal processing in biosensors, while its flexibility allows integration into conformable wearable devices. CNTs, with their extraordinary tensile strength and electron mobility, enhance the durability and sensitivity of strain sensors used for monitoring body movements. Fullerenes, due to their antioxidant properties and reactivity, are utilized in drug delivery systems embedded in wearable devices. CQDs, with their tunable optical properties, excel in fluorescence‐based biosensing for detecting glucose levels, SpO_2_, or cancer biomarkers. These carbon‐based NMs, with their unique nanostructures and bonding characteristics, enable high performance, sensitivity, and stability in wearable health and electronic devices. Their ability to combine mechanical strength, electrical conductivity, and biocompatibility directly supports diverse applications, from physiological monitoring to advanced therapeutic delivery, improving personalized health technologies.

### 2.4 Structural Functionalization

Structural functionalization involves nanoscale engineering to modify and enhance the optical, chemical, and physical properties of photonic NMs, playing a crucial role in the development of advanced wearable sensors, including self‐powered devices. PCs are periodic structures with nanoscale refractive index variations (100–500 nm intervals), creating photonic bandgaps where certain light wavelengths cannot propagate.^[^ ^54^ ^]^ By controlling these bandgaps, PCs enable precise manipulation of light for applications such as optical filters, smart displays, and colorimetric sensors. These devices visually respond to changes in temperature, pressure, or chemical composition. PCs are also employed in tunable lasers and photonic integrated circuits, offering compact solutions for optical communication and health monitoring.^[^ ^2^, ^5^, ^55^ ^]^ Plasmonic nanostructures (PNs) leverage surface plasmon resonance (SPR), where free electrons on metal surfaces resonate with light at specific wavelengths (400–800 nm), creating intense localized electromagnetic fields.^[^ ^56^ ^]^ PNs, including Au and Ag NPs, nanorods, and nanoholes, enhance light absorption and scattering, making them ideal for biosensors and sweat analysis.^[^ ^2^, ^23^, ^28^, ^57^ ^]^ Nanogratings are periodic grooves or ridges (100–500 nm intervals) that manipulate light through diffraction and interference, enabling selective reflection or diffraction of specific wavelengths.^[^ ^58^ ^]^ The nanostructures are widely utilized in wearable devices for environmental monitoring and physiological response detection. Nanogratings integrated into sensors can shift diffraction patterns or produce color changes visible to the naked eye, in response to stimuli such as temperature, pressure, or chemical changes.^[^ ^59^ ^]^ Metamaterials or metastructures, engineered with nanoscale and microscale patterns, exhibit electromagnetic properties not found in nature, such as negative refractive index and superlensing.^[^ ^60^ ^]^ Their structural properties allow for precise control over light propagation, absorption, and reflection.^[^ ^61^ ^]^ In wearable health applications, metamaterials facilitate ultra‐sensitive sensors, high‐resolution imaging systems, and advanced optical communication. Superlensing metamaterials enable imaging at nanoscale resolutions for detecting cellular abnormalities, while flexible metamaterials are developed for skin‐like devices to monitor heart rate and blood oxygen with high accuracy.^[^ ^61^, ^62^ ^]^

Structural functionalization can involve hierarchical designs that combine multiple nanoscale patterns to enhance the sensing performance of wearable devices. Hybrid structures such as PCs combined with plasmonic metal NMs amplify light‐matter interactions, achieving ultrahigh sensitivity and wavelength selectivity. This allows wearable devices to detect trace amounts of biomarkers in bodily fluids like sweat, enabling early disease detection and personalized health monitoring. The wavelength selectivity of these structures also facilitates precise, multi‐wavelength sensing for simultaneous monitoring of different physiological parameters, such as SpO_2_ and hydration levels, without cross‐interference. Similarly, integrating nanogratings with metamaterials creates multifunctional optical devices capable of simultaneous sensing and imaging. These properties are critical for developing compact, high‐performance wearable systems. Nanogratings improve the resolution and sensitivity of optical sensing, resulting in precise detection of physiological changes such as localized inflammation or biomarkers in wound healing. Metamaterials, with the ability to manipulate light at the nanoscale, provide ultra‐sensitive platforms for detecting subtle variations in vital signs or biochemical signals. Such multifunctional systems are particularly advantageous for long‐term, continuous health monitoring applications, including prompt cardiovascular assessment or tracking of chronic conditions such as diabetes or hypertension.

## 3 Light‐Matter Mechanisms, Syntheses, and Functionalizations

This section explores photonic NMs through four key aspects: i) light‐matter interaction properties, ii) sensing and therapeutic modalities, iii) engineering photonic NMs, and iv) functionalization. Specifically, the fundamental principles of light‐matter interactions in photonic NMs are discussed, with a focus on their ability to manipulate light at the nanoscale, enabling advanced sensing and therapeutic modalities for wearable health applications. Fabrication techniques are delved into, including top‐down and bottom‐up engineering approaches, which allow both precision and scalability in device production. Finally, functionalization strategies aimed at enhancing the biocompatibility and optical performance of photonic NMs are presented, ensuring their seamless and effective integration into wearable systems. Each aspect is examined with an emphasis on its distinct working principles, synthesis techniques, and functionalization strategies, highlighting their roles in advancing wearable health devices.

### 3.1 Light‐Matter Interaction Modes and Mechanisms

**Figure 3:**
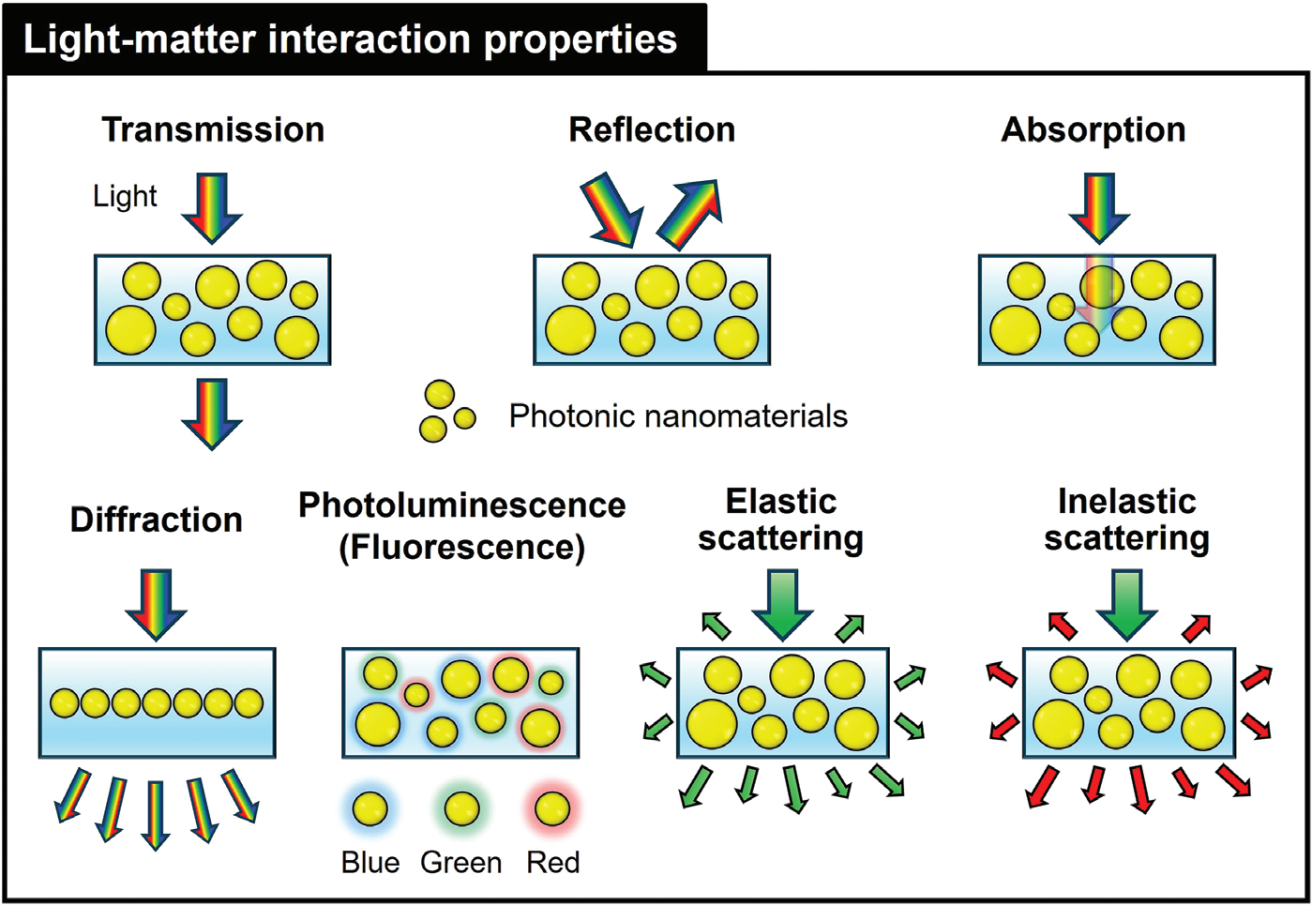
Light‐matter interaction modes and mechanisms of photonic nanomaterials used for wearable health applications: Transmission, reflection, absorption, diffraction, photoluminescence (fluorescence), and elastic and inelastic scattering.

Photonic NMs exhibit diverse mechanisms and modes of light‐matter interactions that can be specifically tailored for diagnostics, monitoring, and treatment in wearable health devices, including skin patches and contact lenses. Light transmission, reflection, absorption, scattering, diffraction, PL (fluorescence), and inelastic scattering play pivotal roles in enhancing the functionality and sensitivity of wearable health technologies for real‐time diagnostic and therapeutic performance (**Figure** **3**).

#### 3.1.1 Transmission and Reflection

Optical transmission and reflection are the fundamental modes of light‐matter interactions that significantly influence the performance of photonic NMs in wearable devices designed for skin and eye applications. Transmission refers to the passage of light through a material, which is determined by its refractive index and absorption characteristics. In wearable health devices, efficient light transmission enables clear vision, precise sensing, selective wavelength filtering, energy efficiency, and enhanced user comfort. High optical transmission can be achieved using transparent, stretchable materials, such as metal nanofibers/nanowires (NFs/NWs), metal NPs, semiconductor thin films, and nanocomposites with Au and Ag NPs for tailored light filtering and protection.^[^ ^63^, ^64^, ^65^, ^66^ ^]^ Substrates with refractive indices optimized to match the photonic NM layer enhance transmittance while minimizing haze, ensuring both comfort and functionality.^[^ ^67^ ^]^ Reflection occurs when light bounces off a material's surface due to refractive index mismatches at the interface. This property enhances optical signal visibility and readability by efficiently capturing light and minimizing energy loss, thereby improving data acquisition in wearable sensors. Reflective photonic NMs and nanostructures are widely applied in wearable devices to achieve such benefits. Plasmonic NP superlattices are used for their stopband features, while PCs such as chain‐like nanostructures, colloidal polystyrene NPs, Au‐relief nanogratings, and silica nanospheres enable real‐time colorimetric monitoring.^[^ ^68^, ^69^, ^70^, ^71^, ^72^ ^]^ Additionally, reflective multi‐nanolayer structures provide precise control and guidance of light, facilitating non‐invasive, battery‐free monitoring.^[^ ^65^, ^73^ ^]^ These systems allow the naked‐eye detection of biomarker concentrations in sweat, enhancing user convenience and accessibility in health applications.

#### 3.1.2 Absorption

Optical absorption refers to the process by which a material captures light energy, initiating electronic transitions or converting it into thermal energy. In wearable devices, the absorption properties of photonic NMs are critical for surface‐interaction‐based, real‐time diagnostics, monitoring, and therapeutic applications. These properties facilitate energy transfer, photodetection, and the generation of heat or ROS. Metal‐based NMs, such as Au and Ag, exploit SPR to enhance light absorption, increasing sensitivity for biomarker detection.^[^ ^74^ ^]^ The photoelectric conversion capabilities of NMs in wearable optical sensors enable efficient light‐to‐electricity conversion, offering high sensitivity across a broad spectral range. These features support real‐time monitoring of vital signs, including heart rate, blood pressure, oxygen levels, pulse oximetry, and respiratory rate.^[^ ^73^, ^75^ ^]^ The ability to convert absorbed light into localized heat is a cornerstone of PTT, while the absorption of specific wavelengths enables photodynamic therapy (PDT) by generating ROS for targeted treatments.^[^ ^74^, ^76^, ^77^ ^]^ Moreover, carefully engineered absorption characteristics can protect eyes from harmful light exposure, enhancing safety in wearable health applications.^[^ ^66^, ^74^ ^]^

#### 3.1.3 Diffraction

Optical diffraction in photonic NMs describes the bending, spreading, or deviation of light as it interacts with nanoscale structures. This phenomenon arises from the periodic arrangement of nanostructures that are comparable in size to the wavelength of visible light.^[^ ^78^ ^]^ Diffraction enables highly sensitive monitoring, diagnosis, and treatment in wearable devices by utilizing diffractive NMs, such as PCs (e.g., nanoprisms, nanogratings, colloidal nanospheres, and thin‐film multilayers), to assess health parameters with precision.^[^ ^79^ ^]^ These nanostructured diffraction patterns detect subtle changes in corneal curvature, intraocular pressure (IOP), skin elasticity, and hydration by sensing light shifts caused by variations in eye or skin properties.^[^ ^80^ ^]^ Diffractive nanostructures are instrumental in diagnosing conditions such as glaucoma, corneal deformations, and skin disorders by analyzing light interactions with biological surfaces.^[^ ^72^, ^80^ ^]^ Additionally, wearable sensors utilizing diffractive NMs support therapeutic monitoring for treatments addressing IOP, corneal irregularities, wound healing, and chronic skin conditions.^[^ ^67^, ^69^, ^81^ ^]^ This capability allows for timely adjustments in care, ensuring optimized outcomes in real‐time health applications.

#### 3.1.4 Photoluminescence

PL refers to the emission of light from a material after absorbing photons, typically resulting from electronic relaxation. PL, including fluorescence, Förster resonance energy transfer (FRET) fluorescence, and electrochemiluminescence (ECL), is a key feature of photonic NMs such as QDs, 2D TMDCs, metal NMs, and upconversion NMs.^[^ ^82^ ^]^ PL is widely utilized in diagnostic, monitoring, and therapeutic applications in wearable devices, where materials emit distinct fluorescence signals under specific light exposure. Fluorescent NMs enable real‐time monitoring of biomarkers in tear fluid, hydration levels, pH, and other physiological parameters, offering critical insights into ocular and systemic health.^[^ ^67^, ^83^ ^]^ In diagnostics, they aid in detecting conditions such as dry eye syndrome and ocular infections.^[^ ^83^, ^84^ ^]^ FRET‐based fluorescence, which relies on energy transfer between two light‐sensitive NMs, improves sensitivity in detecting metabolic imbalances and early indicators of dehydration or skin infections.^[^ ^83b,c,f^ ^]^ Additionally, PL‐based NMs support tracking the effectiveness of therapies, such as treatments for IOP and infections, facilitating real‐time monitoring and personalized adjustments to optimize therapeutic outcomes.^[^ ^65^, ^67^, ^83^ ^]^

#### 3.1.5 Elastic and Inelastic Scattering

Elastic scattering including Rayleigh and Mie scattering is crucial for light interaction without energy change.^[^ ^85^ ^]^ Rayleigh scattering occurs in particles much smaller than the wavelength of light, resulting in wavelength‐dependent scattering useful for sensing molecular interactions, while Mie scattering applies to particles closer to the wavelength size, producing more complex patterns and enabling effects, such as LSPR. Photonic metal NMs make elastic scattering valuable for applications in wearable health devices, such as sensing, where size, shape, and material can be tailored for enhanced optical responses.^[^ ^85^ ^]^ On the other hand, inelastic scattering occurs when incident light exchanges energy with a material, resulting in a shift in the energy of the scattered light, with Raman scattering being a prominent method.^[^ ^86^ ^]^ Raman scattering includes Stokes scattering, where scattered light has lower energy, and anti‐Stokes scattering, where it has higher energy. Due to the low sensitivity of Raman scattering, the SERS technique is widely used for enhancing the Raman signal by adsorbing analytes onto plasmonic metal NMs.^[^ ^87^ ^]^ In wearable devices, plasmonic NPs using SERS are utilized for high sensitivity and trace‐level detection in non‐invasive monitoring and diagnostic applications, including glucose and IOP monitoring and biomarker detection.^[^ ^80^, ^88^ ^]^

### 3.2 Nonlinear Optical Properties

The nonlinear optical properties of photonic NMs can further be used for sensing and therapeutic modalities, including quantum confinement, SPR, Raman scattering, photodetection, fluorescence, FRET fluorescence, upconversion fluorescence, and ECL for sensing and PTT and PDT for therapeutics (**Table** **1**). Integrating photonic NMs enhances the ability to deliver, manipulate, and detect light, providing highly sensitive, real‐time monitoring, diagnosis, and treatment in wearable health devices, including skin patches and contact lenses.

**Table 1:** Sensing and therapeutic modalities of photonic nanomaterials used in wearable devices, including skin patches and contact lenses.

Photonic nanomaterials	Nonlinear optical properties	Light‐matter interaction mode and mechanism	Purpose	Sensing analytes or treated stimuli	Wearable devices	Refs.
Graphene sensitized PbS QDs	Quantum confinement, photodetection	Transmission, reflection, absorption	Monitoring	Heart rate, arterial oxygen saturation (SpO_2_), respiratory rate	Skin patch	[75a]
GQDs	Quantum confinement, FRET	PL	Treatment	Epidermal growth factor (EGF), insulin	Skin patch	[83b]
GQDs	Quantum confinement	PL	Treatment	Latanoprost (anti‐glaucoma drug)	Contact lens	[83d]
Au/Ag nanocomposites	SPR	Transmission, absorption	Treatment	Blue‐light filtering	Contact lens	[66a]
Au NPs	SPR	Absorption	Diagnosis	Exosomes	Contact lens	[96]
Au hollow NWs	SPR	Transmission, absorption	Monitoring, treatment	IOP, timolol	Contact lens	[74d]
Au nanobowls, SiO_2_ NPs	SERS	Absorption, diffraction, inelastic scattering	Monitoring	IOP, MMP‐9	Contact lens	[80g]
Au NWs, Ag nanorods	SERS	Absorption, inelastic scattering	Monitoring	Glucose	Contact lens	[88a]
Au NPs	SERS	Inelastic scattering	Monitoring	Sweat pH	Skin patch	[88c]
Ag NWs coated with 4‐mercaptophenyl boronic acid (MPBA)	SERS	Inelastic scattering	Monitoring	Glucose	Contact lens	[88e]
Ag nanomushroom arrays	SERS	Absorption, inelastic scattering	Monitoring	Urea, lactate, pH	Skin patch	[88g]
Au nanorods	SERS	Inelastic scattering	Monitoring	Glucose, lactate	Skin patch	[88h]
Au NPs	SERS	Absorption, inelastic scattering	Monitoring	Glucose, pesticides (thiram)	Skin patch	[88i]
Au nanosphere cone array	SERS	Absorption, inelastic scattering	Monitoring	Acetaminophen	Skin patch	[88j]
Au NP superlattice	SERS	Absorption, inelastic scattering	Monitoring	pH, lactate content of sweat	Skin patch	[99]
Ag/Au bimetallic inverted nanopyramids	SERS	Absorption, inelastic scattering	Monitoring	Hemoglobin, urea, lactic Acid	Skin patch	[100a]
Au nanostars	SERS	Absorption, inelastic scattering	Monitoring	Lactate, urea, glucose	Skin patch	[100b]
MoS_2_ monolayer	Photodetection	Absorption	Monitoring, diagnosis	Glucose, corneal temperature	Contact lens	[65b]
CdTe QDs	Fluorescence (color change)	Absorption, PL	Monitoring	Fluoroquinolone antibiotics	Ocular eye patch	[83g]
Carbon QDs	Fluorescence (color change)	Absorption, PL	Monitoring	Wound pH	Skin patch	[103]
GQDs	FRET	PL	Treatment	Epidermal growth factor (EGF), insulin	Skin patch	[83b]
MoS_2_ nanosheets	FRET	Absorption, PL	Monitoring	Cortisol	Skin patch	[83c]
Core–shell NaYF_4_:Yb,Er/Tm@ NaYF_4_ UCNPs	Upconversion fluorescence	Absorption, PL	Monitoring	Urea	Skin patch	[83e]
NaGdF_4_:Yb,Er,Ce UCNPs	Upconversion fluorescence	Absorption, PL	Monitoring	Glucose	Skin patch	[83f]
Au nanotubes network with Ru–PEI complex	ECL	PL	Monitoring	Lactate, Urea	Skin patch	[83a]
rGO	ECL	PL	Monitoring	Urea, lactic acid, chloride ions	Skin patch	[110]
Melanin NPs	PTT	Absorption	Treatment	Tumor cells eradication, ROS scavenging, PTT	Skin patch	[76b]
Nanoporous Au ring	PTT	Absorption	Treatment	Residual lens epithelial cells, PTT	Intraocular lens	[76c]
MOFs (CuO_x_@MIL‐101)	PDT	Absorption	Treatment	Scar fibroblast, collagen, apoptosis	Skin patch	[77b]

#### 3.2.1 Quantum Confinement

**Figure 4:**
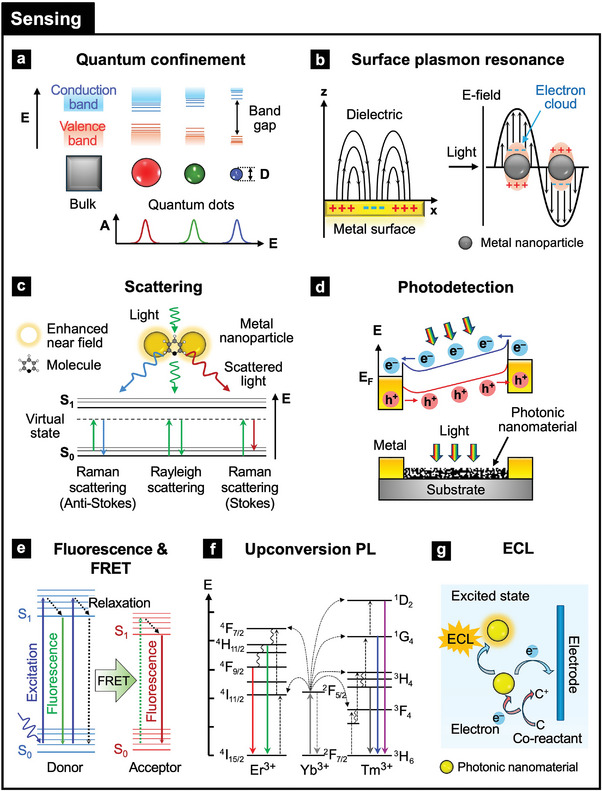
Nonlinear optical properties of photonic nanomaterials for sensing in wearable health devices: a) Quantum confinement, b) surface plasmon resonance (SPR), c) scattering (Rayleigh and Raman), d) photodetection, e) photoluminescence (PL) and Förster resonance energy transfer (FRET), f) upconversion PL, and g) electrochemiluminescence (ECL).

Quantum confinement occurs when the size of a material is reduced to the nanoscale, typically less than the exciton Bohr radius, leading to quantized energy levels (**Figure** **4**a). This phenomenon makes nanoscale quantum materials highly sensitive to environmental changes, including interactions with biomarkers in wearable devices.^[^ ^89^ ^]^ The small size and high surface‐to‐volume ratio of quantum materials enhance interactions with target molecules, increasing sensing sensitivity.^[^ ^90^ ^]^ Precise control of quantum materials’ dimensions during fabrication affects their optical properties, which can be tailored for specific sensing applications in wearable devices.^[^ ^91^ ^]^ This tunability allows for the detection of minute quantities of target biomarkers by lowering detection limits.^[^ ^90^ ^]^ Lead sulfide (PbS) colloidal QDs enhance the sensitivity performance of a graphene‐based photodetector by allowing precise control over light absorption, which enables continuous, low‐power tracking of vital health signs noninvasively such as heart rate, arterial SpO_2_, and respiratory rate.^[^ ^75a^ ^]^ Graphene QDs (GQDs) exhibit improved fluorescence properties, resulting in a ratiometric color shift from red to blue upon oxidation, in response to ROS levels (i.e., hydrogen peroxide, H_2_O_2_) in diabetic wounds.^[^ ^83b^ ^]^ The ratiometric fluorescence, driven by quantum confinement effects, facilitates GQDs to function both as a visual indicator of wound healing status and as a controlled drug‐release mechanism, responsive to oxidative stress within the wound environment. In addition, the enhanced fluorescence properties and responsive “on‐off‐on” fluorescence mechanism of GQDs are utilized to track and visualize drug release in response to enzymatic stimuli, enabling targeted therapeutic delivery.^[^ ^83d^ ^]^

#### 3.2.2 Surface Plasmon Resonance

SPR and LSPR are optical phenomena arising from the interaction of light with metals, providing powerful tools for highly sensitive biomarker monitoring in wearable devices (Figure 4b). SPR occurs at a metal‐dielectric interface, typically on a thin metal film, where surface plasmons oscillate in response to incident light, while LSPR involves the collective oscillation of free electrons in noble metal NPs in response to light.^[^ ^56^, ^92^ ^]^ The high sensitivity of SPR and LSPR comes from their specific absorption wavelengths, which shift in response to environmental changes around the sensor surface, allowing precise detection of biomolecular interactions.^[^ ^93^ ^]^ This resonance shift, rather than intensity changes, enables accurate monitoring of glucose level, hydration, and drug concentrations in biological tissues such as the skin or eyes, along with the detection of infection or inflammation.^[^ ^94^ ^]^ Thus, SPR and LSPR are ideal for real‐time health tracking, supporting personalized diagnostics and treatment by detecting subtle variations in biomarker levels and physiological conditions in wearable health applications.

Au relief nanogratings, which induce surface plasmon polariton modes, are used to enhance sensitivity in a plasmonic‐photonic hybrid waveguide for real‐time humidity monitoring, breathing rate sensing, and skin‐humidity‐based noncontact switching.^[^ ^71^ ^]^ Au nanoshells coated with hydrogel are used to enhance protein recognition in a biosensor for dry eye biomarkers, with LSPR facilitating the detection of lysozyme and lactoferrin in human tears.^[^ ^95^ ^]^ Ag NPs integrated into poly(2‐hydroxyethyl methacrylate) (PHEMA) contact lenses enhance light absorption and filtering for blue‐yellow color vision deficiency, with SPR improving color distinction by filtering out problematic wavelengths.^[^ ^64b^ ^]^ LSPR of Au/Ag NPs in contact lenses enables strong light absorption, enhancing color vision for colorblind patients, filtering blue light to protect the eyes from harmful rays, and providing antibacterial properties to improve eye health and prevent infections.^[^ ^74c^ ^]^ LSPR is employed in the detection of exosomes from tears using Au NPs tagged with antibodies in PHEMA contact lenses.^[^ ^96^ ^]^ This non‐invasive method enables cancer diagnosis and differentiation of exosome surface proteins, enhancing the diagnostic capabilities of contact lenses. SPR based on Au hollow NWs is used in a highly sensitive IOP sensor for continuous IOP monitoring, on‐demand drug delivery, and real‐time intervention in glaucoma treatments.^[^ ^63d^ ^]^

#### 3.2.3 Scattering

Scattering NMs are widely used in wearable devices to enhance the detection of biomarkers for monitoring, diagnosis, and treatment. By utilizing elastic and inelastic scattering, the devices achieve high sensitivity for non‐invasive health monitoring. Elastic scattering, such as Rayleigh or Mie scattering in NMs, occurs when incident light interacts with biomolecules without any changes in the energy of light (Figure 4c). Rayleigh scattering in NMs primarily enhances the sensing ability by enabling the sensitive detection of biomolecules and aids therapy by monitoring drug delivery and cellular responses, thereby supporting more targeted treatments.^[^ ^85^, ^97^ ^]^ In wearable devices, however, inelastic scattering, specifically Raman scattering, is more commonly used than elastic scattering. Raman scattering involves energy shifts (i.e., Raman shift) in light due to molecular vibrations, with Stokes scattering (lower energy, longer wavelength) more common because molecules are generally in their ground state, while Anti‐Stokes scattering (higher energy, shorter wavelength) occurs less frequently but increases with temperature. SERS in NMs is a powerful technique that amplifies the Raman scattering signal of molecules adsorbed on metal NPs, such as Au or Ag.^[^ ^86^, ^87^, ^98^ ^]^ This enhancement results from LSPR, which concentrates electromagnetic fields around the NP ‘hot spots’, significantly increasing the Raman signal of nearby biomarker molecules. SERS is widely applied for diagnostics and monitoring due to its high sensitivity and ability to detect molecules at very low concentrations.

SERS‐based Au nanobowls enhance the detection sensitivity of biomarkers, specifically matrix metalloproteinase 9 (MMP‐9) in tears, enabling precise monitoring of eye health through a smart contact lens.^[^ ^80g^ ^]^ 3D Au/Ag nanostructures are used to amplify Raman signals for the detection of low glucose concentrations in contact lenses.^[^ ^88a^ ^]^ SERS enhances the detection of sweat pH levels by increasing Raman signal sensitivity through Au‐coated polyurethane (PU) NFs functionalized with pH‐responsive molecules (4‐MBA and 4‐MPy), which are integrated into a wearable dermal patch.^[^ ^88c^ ^]^ SERS‐based contact lenses made of silk fibroin and 4‐mercaptophenyl boronic acid (MPBA)‐coated Ag NWs have been developed for selective glucose detection in human tears.^[^ ^88e^ ^]^ SERS from Ag nanomushroom arrays amplifies Raman signals through intense electromagnetic fields, allowing wearable microfluidic sensors to detect biomarkers, such as urea, lactate, and pH levels in sweat.^[^ ^88g^ ^]^ Core–shell structured Au nanorods enhance weak Raman signals to monitor metabolic and hydration levels by detecting glucose and lactate in sweat.^[^ ^88h^ ^]^ The 3D particle‐in‐cavity structure of the Au NPs integrated with silk fibroin and anodic aluminum oxide performs SERS‐based glucose monitoring in sweat for diabetes management.^[^ ^88i^ ^]^ The Au nanosphere cone array based on SERS allows highly sensitive detection of molecules (i.e., acetaminophen) in sweat to monitor drug metabolism, offering valuable insights into an individual's metabolic response to medications for drug monitoring.^[^ ^88j^ ^]^ Patterned Au NP superlattice films in skin patches enable stable, high‐performance SERS for non‐invasive detection of biomarkers such as pH and lactate in sweat.^[^ ^99^ ^]^ The bimetallic Ag/Au nanopyramid structures and multibranched Au nanostars, used in wearable SERS patches, amplify Raman signals to provide real‐time monitoring of sweat biomarkers such as urea, lactic acid, and glucose during physical activities, supporting personalized health monitoring and management.^[^ ^100^ ^]^

#### 3.2.4 Photodetection

Photodetection involves converting light into an electrical signal to detect its presence or intensity using photodetectors, such as photodiodes, phototransistors, and photoconductors, to monitor light presence or intensity in wearable devices.^[^ ^101^ ^]^ When light enters NMs within a photodetector, it excites electrons from the valence band to the conduction band, creating photogenerated electrons, which are then directed toward electrodes to generate an electrical response (Figure 4d). Photodetection is used to detect various biomarkers, as specific biological interactions can cause changes in the intensity, wavelength, or other characteristics of light, which a photodetector then converts into an electrical signal for analysis. A contact lens sensor system incorporates field‐effect transistors with a channel of monolayer MoS_2_ as a photodetector, providing enhanced sensitivity to harmful UV light for retinal protection. This sensor system prevents light‐induced retinal damage and supports early diagnosis of eye health issues.^[^ ^65b^ ^]^ The wearable photodetector incorporates graphene combined with PbS QDs for high sensitivity across a wide light spectrum. The PbS QDs provide photoconductive gain, enhancing signal detection for light intensity changes related to vital signs including heart rate and SpO_2_, supporting real‐time fitness and wellness tracking.^[^ ^75a^ ^]^

#### 3.2.5 Fluorescence and Förster Resonance Energy Transfer

Fluorescence is a form of PL that refers to the emission of light by a photonic NM after photon absorption, as electrons return to their ground state, often emitting light at a longer wavelength and lower energy than the absorbed light (Figure 4e). By integrating fluorescent NMs, such as semiconductor/GQDs, UCNPs, PCs, or carbon dots, into wearable devices, biomarkers or physiological changes in the skin or eyes can be detected with superior sensitivity for monitoring and diagnostics.^[^ ^5^, ^15^, ^102^ ^]^ The wearable skin patch using fluorescent PC nanospheres detects cancer markers, such as carcinoembryonic antigen (CEA) and alpha‐fetoprotein (AFP), enabling real‐time monitoring for early diagnosis in point‐of‐care testing.^[^ ^72^ ^]^ Cadmium telluride (CdTe) QDs boost dual‐emission fluorescence, allowing precise color shifts for monitoring fluoroquinolone antibiotics in tears through a wearable ocular sensor patch.^[^ ^83g^ ^]^ CQDs are synthesized to exploit size‐dependent properties that increase fluorescence and facilitate colorimetric shifts, specifically facilitating pH‐sensitive optical responses suitable for wound healing applications.^[^ ^103^ ^]^

FRET fluorescence involves a non‐radiative energy transfer mechanism, in which energy is transferred from a donor molecule to an acceptor molecule through dipole‐dipole interactions. This process occurs when the donor and acceptor are within close proximity, typically 1–10 nm, enabling energy transfer without photon emission (i.e., non‐radiative energy transfer). FRET‐based sensors offer a non‐invasive way to monitor health indicators.^[^ ^104^ ^]^ The GQD‐decorated porous silicon structure utilizes FRET to produce a ratiometric fluorescence shift from red to blue in the presence of ROS (e.g., H_2_O_2_), providing visual wound monitoring and controlled drug release in diabetic wound dressings.^[^ ^83b^ ^]^ The wearable microfluidic origami device utilizes MoS₂ nanosheets with FRET to quench the fluorescence of dye‐labeled aptamers, detecting cortisol in sweat by fluorescence restoration upon cortisol binding for real‐time stress monitoring.^[^ ^83c^ ^]^

#### 3.2.6 Upconversion Fluorescence

Upconversion fluorescence is a process where absorbed low‐energy photons, typically in the NIR range, are converted into higher‐energy emissions, often in the visible or UV range (Figure 4f). Materials demonstrating upconversion fluorescence typically include lanthanide‐doped NPs such as sodium yttrium fluoride (NaYF_4_), yttrium oxide (Y_2_O_3_), and gadolinium oxide (Gd_2_O_3_), often doped with ions including Yb^3+^, Tm^3+^, or Er^3+^, which facilitate the energy transfer and emission process.^[^ ^82^, ^105^ ^]^ Wearable devices utilizing upconversion NMs leverage their high sensitivity for monitoring applications in diagnostics and personalized medicine.^[^ ^106^ ^]^ NaYF_4_, Er/Tm@NaYF_4_ UCNPs are used in a wearable urea sensor patch, emitting green fluorescence under NIR light that shifts to red with urea for clear visual detection.^[^ ^83e^ ^]^ Cobalt oxyhydroxide (CoOOH)‐coated NaGdF_4_ UCNPs are employed in a wearable hydrogel patch for glucose monitoring. Their fluorescence changes triggered by H_2_O_2_ in sweat provide real‐time support for diabetes management.^[^ ^83f^ ^]^

#### 3.2.7 Electrochemiluminescence

ECL is a process that combines electrochemical reactions with luminescence, where light‐emitting molecules (luminophores) undergo redox reactions under an applied voltage to form excited states that emit photons as they return to their ground state (Figure 4g). The intensity of the emitted light correlates with the concentration of the target analyte, enabling sensitive and quantitative analysis. The integration of NMs, such as QDs and metal NPs, enhances ECL systems by improving sensitivity, stability, and versatility in wearable device applications.^[^ ^107^ ^]^ The ECL method offers sufficient sensitivity for detecting common physiological indicators in the absence of intricate sensitizing modification, because of its inherently high sensitivity.^[^ ^108^ ^]^ In ECL systems, a coreactant is often introduced to facilitate the reaction, producing a strong reducing or oxidizing agent that interacts with the luminophore to generate the excited state responsible for light emission.^[^ ^109^ ^]^ This coreactant‐based mechanism is particularly effective in aqueous environments, overcoming the limitations in direct redox reactions of luminophores. The exceptional sensitivity and specificity of ECL make it a powerful tool for detecting biomarkers in diagnostics.

An ECL sensor with Ru–polyethylenimine (PEI) complexes, comprising the luminophore [Ru(dcbpy)_3_]^2+^ and the coreactant PEI in a single molecule, is encapsulated in silica nanospheres (Ru‐PEI@SiO_2_) and assembled on Au nanotube networks within a flexible polydimethylsiloxane (PDMS) substrate.^[^ ^83a^ ^]^ The ECL sensor detects sweat metabolites through redox reaction‐based light signals. The molecularly imprinted polymer coating ensures selective, sensitive detection of lactate and urea, enabling non‐invasive, real‐time monitoring for personalized health management in wearable devices. Reduced graphene oxide (rGO)‐functionalized hydrogels and luminol‐functionalized hydrogels are integrated into a wearable ECL platform for simultaneous motion monitoring and sweat biomarker detection, including urea, lactic acid, and chloride ions.^[^ ^110^ ^]^ Utilizing a closed bipolar electrode system and self‐healing hydrogels, the platform enables real‐time health tracking with high accuracy and flexibility.

#### 3.2.8 Photothermal Therapy

**Figure 5:**
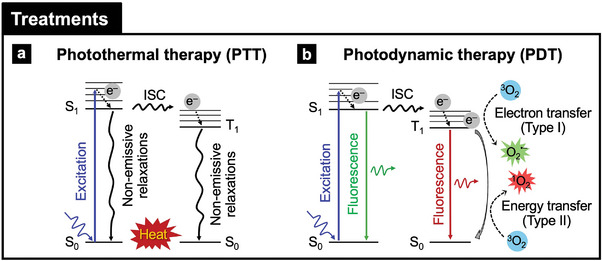
Nonlinear optical properties of photonic nanomaterials for treatments in wearable health devices: a) Photothermal therapy (PTT) and b) photodynamic therapy (PDT). ISC: Intersystem crossing. S_0_: ground sate. S_1_: the lowest singlet excited state. T_1_: triplet state. e^‒^: electrons. ^3^O_2_: molecular oxygen of triplet ground state. O_2_^•‒^: superoxide anion. ^1^O_2_: singlet oxygen.

PTT employs photonic NMs to convert absorbed light into localized heat, effectively targeting and destroying diseased cells.^[^ ^111^ ^]^ In **Figure** **5**a, upon exposure to light, typically in the NIR spectrum, the NM absorbs photons, exciting electrons from the ground state (S_0_) to the lowest singlet excited states (S_1_). Instead of emitting light (e.g., fluorescence) or undergoing intersystem crossing (ISC) to a triplet state (T_1_), the excited electrons return to the ground state through non‐radiative relaxation processes. This relaxation process releases energy as vibrational heat, increasing the local temperature. The localized heat elevates the temperature of the surrounding tissue, leading to the destruction of targeted cells, such as cancerous or infected cells, while also promoting skin wound healing and ocular recovery by eradicating pathogens and enhancing tissue regeneration.^[^ ^76^, ^111^ ^]^

Natural melanin NPs derived from cuttlefish ink (CINPs), known for their antioxidative and photothermal properties, are encapsulated in amorphous silica (SiO₂) shells to enhance their functionality.^[^ ^76b^ ^]^ Encapsulated CINPs in a hyaluronic acid‐based microneedle (MN) patch deliver PTT under NIR light (808 nm), generating heat (51‒56 °C) within 3 min. The patch inhibits tumors, eradicates bacteria, reduces ROS (superoxide anion, O_2_^•‒^), alleviates inflammation, and promotes wound healing. In addition, nanoporous gold (NPG) and other Au nanostructures, including nanorods, nanoshells, and nanocages, are ideal as PTT NMs due to their high photothermal conversion efficiency and biocompatibility.^[^ ^76c^ ^]^ In particular, NPG significantly increases local temperatures (7.0 to 108.2 °C) under NIR light, reducing cell proliferation by 66%. NPG‐based wearable skin patches provide precise, controlled heating (up to 54 °C) for wound healing, hyperplasia control, and inflammation reduction.

#### 3.2.9 Photodynamic Therapy

PDT is a treatment that uses a photosensitizing drug (e.g., molecules) activated by light to target and destroy abnormal cells.^[^ ^19^, ^111^, ^112^ ^]^ PDT involves two key steps: first, administering the photosensitizer, which accumulates in affected tissues, and second, exposing the area to light of a specific wavelength to generate ROS. In Figure 5b, the photosensitization process involves interactions between S_0_, S_1_, and molecular oxygen (O_2_). Through ISC, the T_1_ is formed and can participate in two types of photoreactions. In a Type I photoreaction, T_1_ undergoes electron (e⁻) transfer to the ground state of molecular oxygen (^3^O_2_), generating ROS such as mainly superoxide anion (O_2_^•‒^) and other radicals (H_2_O_2_ and hydroxyl radicals; ‒OH^•^). In a Type II photoreaction, T_1_ transfers energy (E) to ^3^O_2_, producing singlet oxygen (^1^O_2_). Photonic NMs involve themselves or enhance PDT by providing superior light absorption and efficient energy delivery to photosensitizers, enabling targeted treatment of diseased tissues in skin and eye disorders through efficient ROS generation.

Copper oxide (CuO_x_) NPs integrated with porous metal‐organic framework MIL‐101 (CuO_x_@MIL‐101) are used as a photosensitizer in microneedle patches (MNPs) embedded with chloroquine for treating hypertrophic scars.^[^ ^77b^ ^]^ CuO_x_@MIL‐101 generates ROS under visible light (>420 nm), enhanced by autophagy inhibition of chloroquine. Gelatin‐starch MNPs loaded with CuO_x_@MIL‐101 and chloroquine penetrate 300 µm for transdermal delivery. After PDT, fibroblast viability is reduced by 63%, leading to downregulation of TGF‐β1 and Collagen I, which contributes to improved scar management. Titanium dioxide (TiO_2_) NPs in the anatase phase are employed as photosensitizers in a reusable photodynamic transdermal patch for PDT.^[^ ^77c^ ^]^ Activated by blue light (450‒495 nm), TiO_2_ generates both ROS and reactive nitrogen species (RNS), increasing ROS levels 25 fold to eliminate multidrug‐resistant bacteria (i.e., *Escherichia coli*). The patch promotes wound healing, achieving complete closure in 96 h with 95–96% cell viability. Reusable for 20 days with minimal NP release, this cost‐effective, portable device offers antibacterial treatment and wound healing in wearable applications.

### 3.3 Fabrication Engineering

Photonic NMs can have precise control over their structural and optical properties using top‐down and bottom‐up fabrication engineering approaches.^[^ ^113^ ^]^ Top‐down engineering employs techniques such as nanoimprinting, lithography/etching, laser ablation, focused ion beam (FIB), and exfoliation to sculpt nanostructures from bulk materials, achieving desired photonic properties, including enhanced light management. In contrast, bottom‐up engineering involves self‐assembly, chemical synthesis/deposition, and physical deposition to build photonic NMs from the molecular or atomic level, ensuring uniformity and scalability. These fabrication engineering methods enable the fabrication of complex, multifunctional photonic NMs tailored for wearable device applications, enhancing device performance (**Table** **2**).

**Table 2:** Representative top‐down and bottom‐up engineering fabrication methods of photonic nanomaterials for wearable device applications.

Photonic nanomaterials	Engineering approach	Engineering methods	Wearable device	Refs.
PDMS nanopillars	Top‐down	Soft nanoimprint	Contact lens	[80d]
Ag/Au bimetallic inverted nanopyramids		Soft nanoimprint	Skin patch	[100a]
Au hollow NWs		Lithography/etching	Contact lens	[74d]
Ag NW network		Laser ablation	Skin patch	[137]
Diffraction nanogratings		Laser ablation	Contact lens	[80j]
Heart‐shaped Au nanodimers		FIB	Skin patch	[88k]
MoS_2_ monolayer		Au‐mediated exfoliation	Contact lens	[65b]
MoS_2_ nanosheets		Liquid exfoliation	Skin patch	[83c]
Silica NPs	Bottom‐up	Self‐assembly	Skin patch	[67, 80]
Polystyrene NPs		Self‐assembly	Skin patch	[80i]
MOFs (CuO_x_@MIL‐101)		Hydrothermal synthesis	Skin patch	[77b]
Core–shell NaYF_4_:Yb,Er/Tm@ NaYF_4_ UCNPs		Hydrothermal synthesis	Skin patch	[83e]
NaGdF_4_:Yb,Er,Ce UCNPs		Hydrothermal synthesis	Skin patch	[83f]
CdTe QDs		Hydrothermal synthesis	Ocular eye patch	[83g]
Carbon QDs		Hydrothermal synthesis	Skin patch	[103]
Cerium oxide NPs		Hydroxide‐mediated synthesis	Contact lens	[125b]
Ag NPs		Reduction synthesis	Skin patch	[76e]
Bis(10‐hydroxybenzo[h]quinolinato)‐beryllium (bebq2) thin film		PVD	Skin patch	[65a]
Au nanomesh		PVD	Skin patch	[88f]
Au nanosphere cone array		PVD	Skin patch	[88j]
Graphene sensitized PbS QDs		CVD	Skin patch	[75a]
Colloidal QDs, graphene		CVD, hydrothermal synthesis	Skin patch	[124]
Graphene		CVD	Skin patch	[126]

#### 3.3.1 Top‐Down Engineering of Photonic Nanomaterials

**Figure 6:**
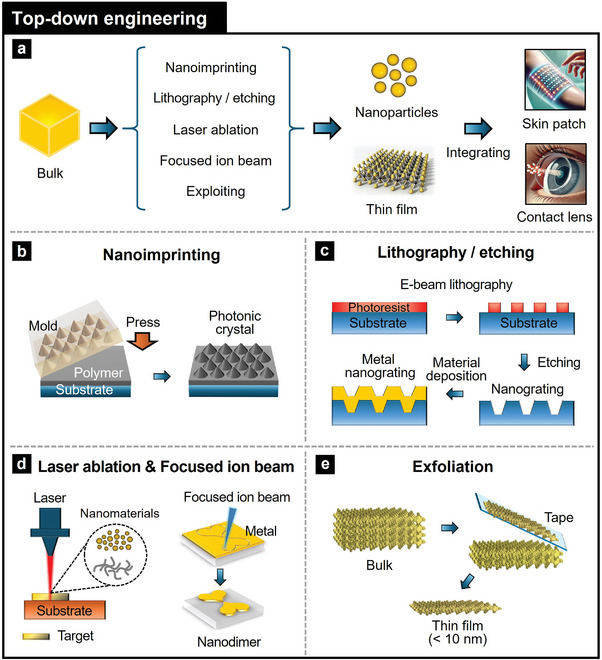
Top‐down engineering processes for the fabrication of photonic nanomaterials. Schematic illustrations of a) overall top‐down engineering process for transforming bulk materials into nanomaterials through techniques such as b) soft nanoimprinting, c) lithography and etching, d) laser ablation and focused ion beam processing, and e) exfoliation for integrating them into wearable health devices (e.g., skin patches and contact lenses). Reproduced with permission.^[^ ^119^ ^]^ Copyright 2020, Wiley‐VCH.

Top‐down engineering fabrication methods offer a systematic approach to transforming bulk materials into photonic NMs with precisely controlled structures and optical properties, making them suitable for producing intricate, high‐resolution features needed in wearable devices (**Figure** **6**a). Nanoimprinting enables rapid, large‐scale patterning, while lithography and etching produce high‐resolution designs.^[^ ^114^ ^]^ Laser ablation^[^ ^115^ ^]^ and FIB^[^ ^116^ ^]^ processing allow for localized modifications to create tailored nanostructures. Exfoliation supports the production of thin, atomic‐scale layers.^[^ ^117^ ^]^ These methods collectively advance the development of wearable devices, such as skin patches and contact lenses, enhancing their functionality and sensitivity in real‐time diagnostics, monitoring, and treatments. Soft nanoimprint lithography is a high‐resolution, large‐area, and cost‐effective nanofabrication technique that replicates nanoscale patterns on the surface of rigid or flexible materials using nanopatterned master molds through thermal or UV curing processes (Figure 6b). This lithography technique is widely used in wearable devices, offering scalability and versatility. Strain‐sensitive nanopillars on stretchable PDMS are fabricated by nanoimprint lithography for scalable, electronics‐free IOP monitoring (15‒35 mmHg) with smartphone‐based optical readouts integrated into contact lenses for glaucoma management.^[^ ^80d^ ^]^ High‐precision Ag/Au‐coated nanopyramids with sharp tips and strong electromagnetic hotspots are fabricated using nanoimprint lithography to detect sweat biomarkers such as urea and lactic acid for health monitoring and personalized diagnostics in wearable SERS sensors.^[^ ^100a^ ^]^

Lithography and etching are critical techniques for fabricating nanoscale structures with high resolution (Figure 6c). Lithographic methods including photolithography, electron beam lithography (EBL), nanosphere lithography, and laser‐interference lithography (LIL) enable precise nanopatterning, with EBL offering sub‐10 nm precision and LIL being effective for large‐area periodic designs.^[^ ^118^ ^]^ Etching techniques, such as reactive ion etching (RIE), inductively coupled plasma etching, chemical wet etching, and metal‐assisted chemical etching, further refine these lithographic nanopatterning to create intricate nanostructures.^[^ ^118^ ^]^ Together, these techniques support the development of photonic functional nanostructures, particularly for applications in wearable devices. A plasmonic‐photonic hybrid system (PPHS) with an Au nanogroove grating (520 nm periodicity, 250 nm depth) and an 880 nm‐thick PVA layer is fabricated using EBL and deep RIE processes.^[^ ^71^ ^]^ The PPHS sensor achieves high sensitivity due to its hybrid optical waveguide and surface plasmon polariton modes. It provides a wavelength shift of 1.16 nm%RH⁻¹ and intensity sensitivity of 1.28%RH⁻¹, enabling the monitoring of physiological parameters such as respiration. Hollow Au NWs, fabricated by selectively etching Ag cores with nitric acid and opening Au channels via RIE, exhibit high visible transmittance, NIR absorbance, and strong SPR effects.^[^ ^63d^ ^]^ The nanostructures are integrated into contact lenses for real‐time IOP monitoring and targeted glaucoma treatment through controlled drug release. In addition, lithography and etching techniques are extensively used to create nanopatterned master molds for soft nanoimprinting, facilitating high‐resolution, flexible nanostructures in wearable devices for applications, such as biosensing, strain detection, and personalized diagnostics.^[^ ^80^, ^100^ ^]^

Laser ablation employs high‐energy laser pulses to precisely remove material from solid surfaces, facilitating the synthesis of various nanostructures, including metal NPs, graphene‐based materials, and core–shell structures (Figure 6d). Laser ablation offers fine control over size and morphology, produces minimal by‐products, and is adaptable to a wide range of materials, making it environmentally friendly and versatile.^[^ ^115^ ^]^ In wearable devices, laser‐ablated NMs or nanostructures are utilized to enhance functionalities such as high transparency, diffraction, and strong SPR, contributing to advancements in personalized health monitoring and diagnostics. Ag NW networks are fabricated using laser‐induced ablation, where a focused laser precisely patterns conductive lines as narrow as 150 µm on Ag NW films while maintaining their structural integrity and electrical conductivity as well as high optical transmittance (92%).^[^ ^63c^ ^]^ Integrating into wearable optoelectronic devices enables simultaneous light delivery and thermal monitoring in a wireless skin patch for applications such as blood flow monitoring, PTT, and PDT. Curved and straight diffraction nanostructures on hydrogel‐based contact lenses are fabricated using holographic laser ablation with a nanosecond Nd:YAG laser (1064 nm), achieving periodicities of 820–860 nm and groove spacings of 473 nm.^[^ ^80j^ ^]^ The nanostructures produce wavelength‐dependent rainbow diffraction with a 4° angle variation under hydrated conditions. Integration of these nanostructures into contact lenses enables non‐invasive monitoring of IOP and potassium ion (K^+^) levels, providing valuable diagnostics for ocular and systemic health. FIB technologies are a powerful nanofabrication tool that utilizes a focused ion beam to precisely manipulate materials at the nanoscale.^[^ ^116^ ^]^ With its high spatial resolution, FIB enables patterning, etching, deposition, and modification of materials without requiring photolithographic masks. Heart‐shaped Au NP dimers with 5 nm nanogaps are fabricated using focused helium ion beam (He^+^‐FIB) milling and “sketch‐and‐peel” lithography, achieving nanoscale precision for enhanced plasmonic performance.^[^ ^88^, ^119^ ^]^ The nanodimers provide SERS enhancement factors up to 10^11^ at a wavelength of 785 nm, with electric field amplification exceeding 600 times near the nanogaps.^[^ ^88k^ ^]^ Integrated into flexible PDMS substrates, formed a wearable skin patch, the nanodimers function as stretchable SERS metasurfaces for real‐time, non‐invasive sweat analysis.

Exfoliation methods create ultra‐thin 2D nanosheets by separating bulk materials, leveraging weakened van der Waals interactions.^[^ ^117^ ^]^ Methods include mechanical exfoliation for high‐quality single layers, chemical exfoliation for large‐scale production with functionalized surfaces, and liquid exfoliation for scalable nanosheet dispersion (Figure 6e). Ultra‐thin MoS_2_ films are fabricated via an Au‐mediated exfoliation process, producing mono‐ to few‐layer flakes (area: 200 × 100 µm, thickness: 1.8 nm for few‐layers) with high surface‐to‐volume ratios and dangling‐bond‐free surfaces.^[^ ^65b^ ^]^ The MoS_2_ films exhibit high optical transparency of ≈93% and a photoresponsivity of 4.8 A W^‒1^ under UV light, making them ideal for wearable photodetectors, glucose sensors, and temperature sensors in contact lenses. MoS_2_ nanosheets are fabricated via liquid exfoliation, producing nanosheets (200‒500 nm) with a high surface area and a preserved 2H polytype structure.^[^ ^83c^ ^]^ The MoS_2_ nanosheets exhibit strong fluorescence quenching through FRET in a skin patch, enabling their use in biosensors. Integrated into a 3D origami microfluidic device, they allow real‐time cortisol monitoring for stress management.

#### 3.3.2 Bottom‐Up Engineering of Photonic Nanomaterials

**Figure 7:**
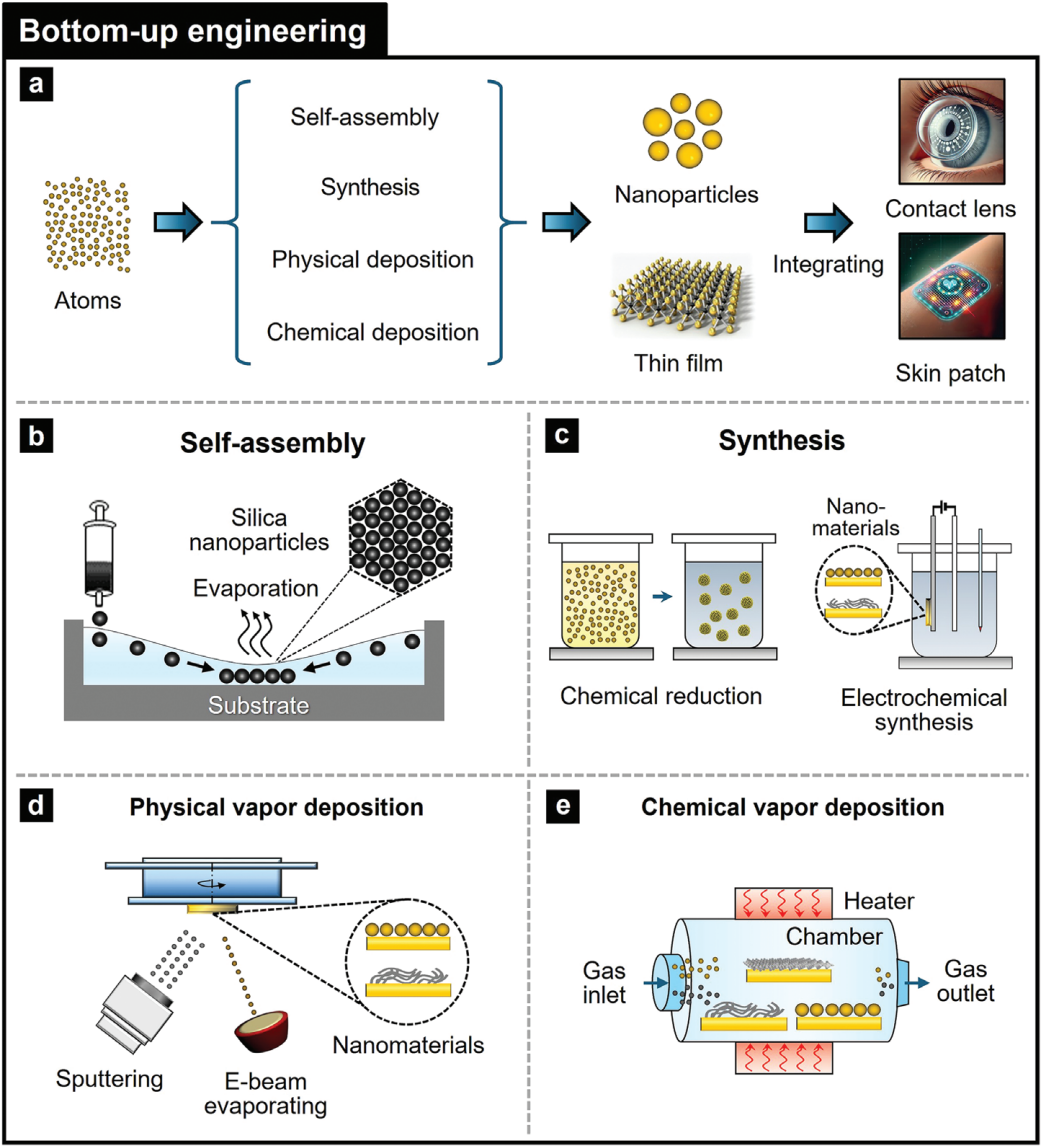
Bottom‐up engineering processes for the fabrication of photonic nanomaterials. Schematic illustrations of a) overall bottom‐up engineering process for transforming atoms or molecules into nanomaterials through techniques such as b) self‐assembly, c) synthesis, d) physical vapor deposition, and e) chemical vapor deposition for integrating them into wearable health devices (e.g., skin patches and contact lenses).

Bottom‐up engineering fabrication techniques assemble atoms or molecules into precisely structured forms to produce photonic NMs (**Figure** **7**a). Self‐assembly uses intrinsic molecular forces to spontaneously organize atoms or molecules into ordered nanostructures.^[^ ^120^ ^]^ Synthesis builds NMs by assembling atoms or molecules through nucleation and growth processes.^[^ ^113^, ^121^ ^]^ Physical vapor deposition (PVD) and chemical vapor deposition (CVD) methods are utilized to form uniform, high‐quality photonic NMs onto substrates.^[^ ^122^, ^123^ ^]^ These techniques advance wearable devices such as skin patches and contact lenses, enhancing their sensitivity, flexibility, and real‐time diagnostic and therapeutic functionalities. Self‐assembly is widely used for creating NMs by arranging nanoscale components using weak interactions, such as van der Waals forces, and external stimuli, such as magnetic or electric fields for precise organization (Figure 7b).^[^ ^120^ ^]^ Silica and polymer NPs form highly ordered structures through capillary forces during solvent evaporation or electrostatic interactions, resulting in periodic nanostructures. Self‐assembly offers scalability, precision, and versatility, making it ideal for applications in wearable devices. The self‐assembly method arranges NPs into periodic ordered structures through various approaches, including evaporation‐induced processes, capillary force‐driven assembly, and electrostatic interactions.^[^ ^67^, ^80^ ^]^ Silica NPs form hexagonal close‐packed templates at gas‐liquid interfaces or in confined spaces, while polystyrene NPs create periodic lattice structures. These ordered nanostructures exhibit tunable optical properties, such as structural colors and reflection peak shifts. The versatility of photonic NPs formed by the self‐assembly method enables applications in real‐time monitoring.

NM synthesis involves nucleation and growth processes facilitated by hydrothermal, reduction, and chemical methods (Figure 7c).^[^ ^113^, ^121^ ^]^ Hydrothermal synthesis relies on high‐temperature and high‐pressure conditions to form well‐structured NMs, allowing the precise fabrication of various NMs such as MIL‐101(Cr) metal‐organic frameworks (MOFs) integrated with CuO_x_ NPs, UCNPs (NaYF_4_ and NaGdF_4_), and QDs (carbon, CdSe/CdS/ZnS, and CdTe).^[^ ^77^, ^83^, ^103^, ^124^ ^]^ These synthesis methods enable precise control over size, morphology, and crystallinity, as well as environmentally friendly and scalable production. The synthesized NMs exhibit optical properties, such as pH‐sensitive and NIR upconversion fluorescence, colorimetric response, photocatalytic ROS generation, supporting wearable applications for wound monitoring, PDT, glucose/urea detection, and antibiotic profiling. Hydroxide‐mediated synthesis is employed for the fabrication of cerium oxide NPs, which are utilized in colorimetric contact lenses for non‐invasive glucose monitoring in tears.^[^ ^125^ ^]^ This method takes advantage of cerium's reversible oxidation states (Ce^3+^ and Ce^4+^) to generate distinct color changes in response to glucose levels. The reduction approach employs chemical agents to generate metallic NPs with tailored properties. Ag NPs are synthesized through the reduction of Ag ions, with bovine serum albumin serving as a stabilizing agent.^[^ ^76e^ ^]^ In addition, NM synthesis is the controlled process of combining simpler substances to form NMs with specific structures and properties through chemical reactions.^[^ ^83a^ ^]^ Au nanotube networks are chemically synthesized using Au(en)_2_Cl_3_ ((bis(ethylenediamine)gold(III) chloride) as a precursor, replacing Ag NWs on a PDMS substrate. Enhanced with Ru‐PEI@SiO_2_ nanospheres for ECL and coated with molecularly imprinted polymers, they facilitate sensitive sweat metabolite detection for real‐time monitoring in wearable patches. These methods facilitate the synthesis of stable, biocompatible NPs with controlled size and dispersion, making them suitable for wearable applications.

PVD is used to fabricate thin films and nanostructures by vaporizing solid materials and condensing them onto substrates (Figure 7d). PVD methods such as electron‐beam/thermal evaporation, pulsed laser deposition, sputtering, and spin coating provide over film thickness and composition, producing versatile and scalable photonic NMs with high purity, scalability, and flexibility for wearable device applications. A light‐emitting thin film of bis(10‐hydroxybenzo[h]quinolinato)‐beryllium (bebq2) with nanolaminate barrier layers (zinc oxide, ZnO; aluminum oxide, Al_2_O_3_; magnesium oxide, MgO) is deposited on a polyethylene terephthalate (PET) substrate by using thermal evaporation for flexible organic LEDs.^[^ ^65a^ ^]^ These NMs are optimized for wound healing and attachable phototherapeutic applications. Additionally, the Au deposition onto polyvinyl alcohol (PVA) NFs using thermal evaporation results in Au nanomeshes for SERS sensor‐based wearable patches (i.e., sweat biomarkers).^[^ ^88f^ ^]^ Ion sputtering is employed to fabricate Au nanosphere cone arrays by depositing an Au layer onto silicon nanocone arrays, enabling sensitive SERS‐based monitoring of acetaminophen in drug‐administered subjects.^[^ ^88j^ ^]^

CVD is a process in which gaseous precursors undergo chemical reactions in an activated environment, forming a stable solid product that deposits on a heated substrate (Figure 7e). The process involves generating reactive gases, transporting them to a reaction chamber, and enabling surface or gas‐phase reactions to form dense or porous coatings.^[^ ^122^, ^123^ ^]^ It ensures high purity, atomic‐scale control, and uniform deposition on complex shapes due to its non‐line‐of‐sight capability. Key factors such as temperature, pressure, and gas composition determine deposit quality, while specialized reactor designs provide precise control over reactions and substrate heating. GQDs are used in flexible photodetectors for tracking vital signs (i.e., heart rate and SpO_2_) in health‐monitoring patches.^[^ ^75a^ ^]^ Single‐layer graphene synthesized via CVD on copper foil is transferred to substrates for glucose sensing in soft contact lenses and transparent electrodes in stretchable patches, respectively.^[^ ^63^, ^124^ ^]^ Multilayered graphene formed with CVD serves as electrodes in capacitive temperature sensors for wearable thermal patches, supporting applications in wound healing and skin disease diagnosis.^[^ ^126^ ^]^

### 3.4 Functionalization

Functionalization with photonic NMs can further enhance biocompatibility and optimize optical sensing performance.^[^ ^91^, ^105^ ^]^ These materials must ensure safety, comfort, and long‐term wearability while maintaining their functional performance.

#### 3.4.1 Biocompatibility

Biocompatibility is a crucial factor for photonic NMs used in wearable health applications, particularly in devices that come into direct contact with the skin or eyes, such as epidermal patches and contact lenses. Approaches such as surface passivation and polymeric coatings minimize the potential toxicity of photonic NMs.^[^ ^84^, ^127^ ^]^ Such approaches reduce the risk of adverse reactions, including inflammation, irritation, or cytotoxicity, while preserving or enhancing optical and mechanical properties. For example, polymer or silk encapsulation enhances the biocompatibility of photonic NMs such as TiO_2_ and photonic crystal NPs, enabling reusable wound‐healing epidermal patches and 3D‐printed wearable sensors with flexibility, self‐healing, and real‐time diagnostic capabilities.^[^ ^72^, ^77^ ^]^ Structurally colored contact lens sensors made from PHEMA hydrogel and MoS_2_‐based smart contact lenses encapsulated with polyimide demonstrate biocompatibility through cytotoxicity tests and in vivo assessments, enabling reliable, continuous ocular health monitoring.^[^ ^65^, ^80^ ^]^

#### 3.4.2 Optical Performance

First, functionalization with photonic NMs improves the sensing performance of wearable devices. QDs functionalized with PEG or silica achieve stabilized fluorescence, while rare‐earth UCNPs modified with CoOOH or silica shells enhance luminescence and prevent dissolution.^[^ ^83^, ^84^, ^127^ ^]^ Embedding NMs in hydrogels provides highly‐responsive optical biosensing, including ion detection (Fe^2+^, Cu^2+^, Hg^2+^) and H_2_O_2_ monitoring for glucose oxidation.^[^ ^83^, ^127^ ^]^ Functionalized NMs significantly improve the sensitivity and versatility of wearable health devices. Sweat sensors utilize superhydrophilic surfaces or stretchable substrates to enhance fluid capture, while SERS sensors with nanostructured coatings detect biomarkers (e.g., pH, chloride, glucose, calcium, ammonia, lactate, ethanol, and urea levels) in sweat with high specificity.^[^ ^100^, ^128^ ^]^ Drug delivery systems, incorporating GQDs and mesoporous silica, combine theragnostic with fluorescence imaging for targeted treatments.^[^ ^80^, ^83^ ^]^ Second, stimuli‐responsive functionalization, including (e.g., ROS, pH, glucose), physical (e.g., light, heat, pressure), and biological (e.g., enzymatic activity) triggers is highly beneficial for advanced wearable health technologies.^[^ ^91^, ^105^, ^129^ ^]^ Oxidation‐triggered fluorescence recovery for glucose monitoring and ROS‐responsive drug release for diabetic wound healing enables real‐time, personalized health.^[^ ^83b,f^ ^]^ Wearable hydrogel patches embedded with functionalized NPs precisely monitor physiological markers (e.g., sweat glucose, H_2_O_2_, and electrolyte levels), through distinct color changes.^[^ ^80^, ^83^, ^130^ ^]^

## 4 Device Assembly of Photonic Nanomaterials for Wearable Health Applications

Photonic NMs enable significant advancements in device form factors, sensing capabilities, energy efficiency, and data communication for wearable health applications. Their integration facilitates the development of ultra‐thin, flexible, and stretchable devices that conform seamlessly to the body, enhancing both comfort and functionality. These NMs enable real‐time monitoring of vital signs and biomarkers, providing continuous and accurate health insights. Self‐powered systems incorporating photonic NMs reduce reliance on external energy sources, ensuring greater autonomy. Additionally, wireless communication systems in wearable devices enable reliable and efficient transmission of health data for timely analysis and intervention. This section explores the assembly of photonic NM‐based devices for wearable health applications, focusing on three key aspects: i) device form factors, ii) sensing capabilities, and iii) power and data communication methods. Each aspect is analyzed with an emphasis on strategies for integrating photonic NMs seamlessly into wearable health systems.

### 4.1 Device Formfactors

Photonic NMs demonstrate exceptional feasibility for wearable device applications due to their ability to achieve ultra‐thin, stretchable, and flexible formfactors. The NMs facilitate seamless integration with human skin or eye surfaces, maintaining comfort and performance during movement. Ultra‐thin structure of NMs minimizes weight and ensures unobtrusive wearability, while stretchability and flexibility allow for conformal contact and durability under mechanical deformation. Ultra‐thin, flexible, and stretchable form factors for photonic NMs in wearable devices can be achieved through a combination of advanced fabrication methods, intelligent design strategies, and machine learning‐assisted design approaches. First, top‐down engineering methods, such as nanoimprinting, lithography, and laser ablation, facilitate the formation of high‐resolution and precise nanostructures and NMs, while exfoliation processes yield ultra‐thin NMs (e.g., MoS_2_, WS_2_, and graphene) to enhance flexibility and minimize mechanical stiffness.^[^ ^65^, ^114^, ^115^, ^131^ ^]^ Second, bottom‐up engineering methods, including self‐assembly, synthesis, and vapor deposition (i.e., CVD, PVD), support scalable production of ultra‐thin, flexible, and stretchable NMs with controlled mechanical and optical properties.^[^ ^88^, ^113^, ^120^, ^121^, ^128^ ^]^ Third, intelligent design‐enabling processes (e.g., deposition, transfer printing, encapsulation, and embedding) can facilitate the integration of photonic NMs with flexible and stretchable polymers.^[^ ^65^, ^88^, ^124^, ^128^, ^132^ ^]^ Finally, Machine‐based design principles can aid in achieving ultra‐thin, flexible, and stretchable form factors for NMs in wearable devices.^[^ ^133^ ^]^ These factors collectively make photonic NMs a versatile choice for developing advanced wearable health devices, including skin patches and contact lenses.

**Figure 8:**
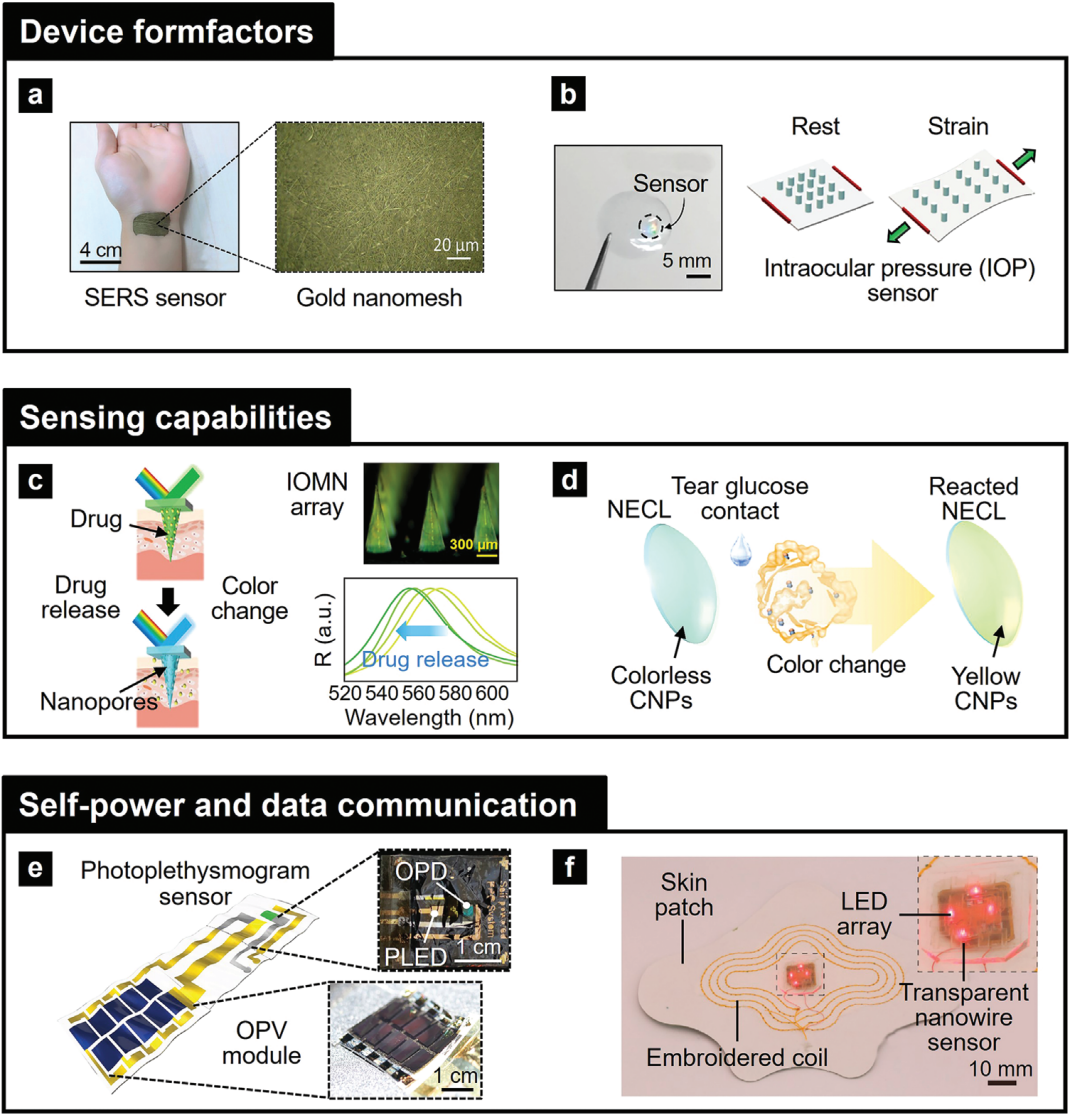
Device assembly of photonic nanomaterials for wearable health applications. (a&b) Device formfactors: ultra‐thin, flexibility, and stretchability. a) Wearable SERS sensor adhered to a human wrist (left) and 50× optical microscopy image of the Au nanomesh (right). Reproduced with permission.^[^ ^88f^ ^]^ Copyright 2022, Wiley‐VCH. b) Stretchable ocular pressure sensors. Photograph of a contact lens integrating an optical strain sensor based on PDMS nanopillars (left) and schematic illustration for the working principle of the strain sensor. The application of stress causes the sensor to stretch, inducing a change in the grating spacing. Reproduced with permission.^[^ ^80d^ ^]^ Copyright 2021, American Chemical Society. c,d) Sensing capabilities for real‐time monitoring and diagnostics. c) Schematic illustration of drug delivery and real‐time self‐monitoring application of the inverse opal microneedle (IOMN) arrays. Photograph of hydrogel IOMN array by replicating the templates silica NPs with diameters of 200 nm. Blue‐shift reflection peak of IOMNs during drug release. Reproduced with permission.^[^ ^67^ ^]^ Copyright 2022, Wiley‐VCH. d) Schematic illustration of colorimetric nanoparticle‐embedded contact lenses (NECLs) for real‐time monitoring of tear glucose. NECL color is changed to yellow (colorless Ce^3+^ to yellow Ce^4+^) as it is exposed to tear depending on glucose concentration. CNPs, cerium oxide NPs. Reproduced with permission.^[^ ^125b^ ^]^ Copyright 2021, American Chemical Society. e,f) Self‐powered and data communication wearable health devices integrated photonic nanomaterials. e) Self‐powered photoplethysmogram sensor. Schematic diagram of the self‐powered photoplethysmograpm (PPG) sensor on human hands. The organic photovoltaic (OPV) module generates electrical power from sunlight and drives polymer light‐emitting diode (PLED) and organic photodiode (OPD). Photographs of the OPV module under one‐sun illumination and self‐powered PPG sensor. Reproduced with permission.^[^ ^75b^ ^]^ Copyright 2021, Springer Nature. f) Wireless optoelectronic patch and integrated transparent sensor. Photograph of the wirelessly powered optoelectronic patch. The inset shows the LED array with an integrated transparent temperature sensor. A transmitter coil operating in the 13.56 MHz industrial, scientific, and medical band is used for a wireless power transfer system. Reproduced with permission.^[^ ^63c^ ^]^ Copyright 2022, Cell Press.

The ultra‐thin structure of photonic NMs is critical to achieving device functionality and seamless integration in wearable devices. SERS sensors utilize ultra‐thin Au nanomesh layers (≈150 nm), enhancing flexibility, adhesion, and molecular detection via nanoscale hotspots (**Figure** **8**a).^[^ ^88f,i^ ^]^ MoS_2_ films with a nanoscale thickness of 1.8 nm (few‐layer structure) for transistor devices reduce mechanical stiffness, enhance transparency, and ensure comfort while providing high‐sensitivity biomarker detection in smart contact lenses.^[^ ^65b^ ^]^ Furthermore, scalable designs reduce material usage while supporting diverse analyte sensing.^[^ ^88f^ ^]^ The flexibility and stretchability of photonic NMs, combined with polymeric materials such as PDMS, PET, and PU, are essential for advancing wearable device technologies, offering critical advantages in comfort, adaptability, and performance. Flexibility enables devices to conform naturally to curved and dynamic surfaces, ensuring stable operation during movement and enhancing real‐time monitoring capabilities. On the other hand, stretchability allows devices to detect mechanical changes, as demonstrated by PDMS‐based diffraction gratings that adjust under strain for precise, real‐time ocular pressure monitoring (Figure 8b).^[^ ^80d^ ^]^ In addition, they facilitate devices to maintain functionality under mechanical deformation, such as stretching, bending, or twisting, ensuring their reliability in dynamic environments.^[^ ^124^ ^]^ Stretchable optoelectronic sensors can sustain up to 70% strain without performance loss, providing continuous health monitoring through photoplethysmography (PPG). Stretchable smart contact lenses adapt comfortably to the eye's curvature, integrating wireless glucose monitoring and data display for a secure fit.^[^ ^63a^ ^]^ In wearable SERS sensors, stretchable metasurfaces preserve Raman signal quality under strain, allowing high‐precision chemical analysis even during movement.^[^ ^88k^ ^]^ Flexible sweat sensors ensure accurate sweat collection and biomarker detection by conforming to skin.^[^ ^128^ ^]^ Flexible OLED photonic skin patches adapt seamlessly to body contours, allowing targeted, non‐invasive therapeutic light delivery for wound healing while maintaining user comfort.^[^ ^65a^ ^]^ Furthermore, flexible substrates in Raman sensors and ECL sensors maintain consistent performance by adapting seamlessly to irregular or uneven body surfaces.^[^ ^83^, ^88^ ^]^

### 4.2 Sensing Capabilities

Photonic NMs offer exceptional sensing mechanisms by leveraging unique light‐matter interaction properties, including absorption, reflection, scattering, and emission. First, such photonic NMs can detect minute biomolecular changes with high sensitivity and precision (Figure 3) and enhance sensing accuracy and responsiveness. Second, NMs can provide enhanced sensing specificity. By integrating nanoscale structures (e.g., QDs, graphene, PCs, and NPs) into wearable sensors, it becomes possible to enable specific sensing applications, including monitoring glucose levels, oxygen saturation, and hydration. This capability allows the detection of critical biomarkers for diseases, including diabetes, glaucoma, and skin cancer. Finally, the multimodal capabilities of photonic NMs enable multiplexing to detect multiple physiological parameters simultaneously. On the skin, QD‐based devices offer precise monitoring of vital signs including blood flow variations through PPG signals, providing real‐time cardiovascular assessments.^[^ ^124^ ^]^ Graphene‐based thermal patches provide high‐resolution temperature mapping and perform thermotherapy for managing wounds and skin conditions, integrating diagnostic and treatment functions seamlessly.^[^ ^126^ ^]^ Furthermore, multifunctional NMs and plasmonic hybrid systems utilize photonic nanostructures to monitor drug release rates and hydration levels, ensuring precise therapeutic interventions (Figure 8c).^[^ ^67^, ^71^ ^]^ For eye applications, NP‐embedded smart contact lenses perform non‐invasive glucose monitoring via tear fluid analysis, with wireless displays offering real‐time insights into diabetes management (Figure 8d).^[^ ^125b^ ^]^

In addition, photonic NMs enable multiplexing wearable devices to detect multiple physiological and biological signals simultaneously. For example, SERS‐based sweat sensors with patterned Au NP superlattice films provide real‐time analysis of sweat volume, pH, and lactate, delivering comprehensive physiological data.^[^ ^99^ ^]^ Smart diapers with raspberry‐shaped Au NPs facilitate simultaneous, label‐free detection of urea, creatinine, and bilirubin through enhanced Raman signals, providing real‐time, non‐invasive monitoring of kidney and liver health.^[^ ^88l^ ^]^ Additionally, hydrogel‐based patches integrate rGO for motion monitoring and luminol‐functionalized c‐BPE systems for sweat analysis, providing the concurrent detection of physical activity and biomarkers (urea, lactic acid, and chloride ions) through ECL.^[^ ^110^ ^]^ Multifunctional contact lenses integrate MoS_2_ transistors, serpentine Au NWs, and photodetectors to monitor glucose, corneal temperature, and light exposure, offering a non‐invasive platform for comprehensive ocular and systemic health monitoring.^[^ ^65b^ ^]^ Dual‐emission fluorescence skin patches combine CdTe QDs and lanthanide ions, leveraging an aggregation‐regulated antenna effect alongside microfluidic sensing and machine learning to simultaneously detect multiple fluoroquinolone antibiotics (e.g., ciprofloxacin, norfloxacin, ofloxacin, and lomefloxacin) in tears.^[^ ^83g^ ^]^

### 4.3 Power and Data Communication Methods

Integrating self‐powered (battery‐free) technologies and advanced data communication systems into wearable health devices addresses industrial and commercial needs for sustainability, scalability, lower maintenance, and longer life.^[^ ^134^ ^]^ The self‐powered wearable devices incorporate energy harvesting systems using piezoelectric, triboelectric, and photovoltaic NMs to eliminate the need for conventional batteries.^[^ ^134^, ^135^ ^]^ Self‐powered systems, such as glucose fuel cells, triboelectric nanogenerators, organic photovoltaic (OPV) cells, and stretchable non‐enzymatic fuel cells, harvest energy from natural sources including body fluids, movement, or ambient light to operate wearable devices without the need for external power (e.g., conventional batteries).^[^ ^75^, ^80^, ^132^, ^134^ ^]^ This enhances device sustainability, reducing maintenance and improving user comfort. The self‐powered systems are exploited in skin‐mounted patches or smart contact lenses, offering continuous monitoring of physiological parameters such as glucose levels, NW, or wound healing progress (Figure 8e).^[^ ^75b^ ^]^ However, energy harvesting in wearable devices faces challenges in energy density and lifespan. AI‐designed photonic NMs, which optimize light absorption and power conversion, offer a solution, particularly in low‐light conditions.^[^ ^136^ ^]^ Hybrid power systems combining photonic NMs with triboelectric nanogenerators and photovoltaic technologies harness both mechanical and light energy for reliable power. AI‐assisted photonic NM and hybrid power systems enable durable, sustainable wearable devices for efficient long‐term health monitoring and treatment.^[^ ^137^ ^]^

The transparency and flexibility of photonic NMs, such as transparent Ag NW networks and nano/microvoid polymers, provide several advantages including efficient real‐time data exchange, robust communication, and enhanced thermal regulation, ensuring reliable wireless performance even in dynamic and outdoor environments.^[^ ^138^, ^139^ ^]^ Data communication technologies, such as near‐field communication (NFC), integrated wireless circuits, and wireless modules, enable real‐time, high‐speed, and interference‐free data transmission, facilitating seamless integration with wearable devices for remote monitoring and diagnostics.^[^ ^63^, ^126^, ^139^ ^]^ Specifically, photonic NM‐based transparent electrodes and stretchable optical fibers enable simultaneous sensing and data transmission without hindering device functionality, while their integration with flexible substrates ensures durability and adaptability under physical strain.^[^ ^63^, ^138^ ^]^ Integrated sensing and display systems further benefit from photonic nanostructures and embedded LEDs, facilitating synchronized health data visualization and real‐time monitoring.^[^ ^63^, ^139^ ^]^ Efficient data communication facilitates non‐invasive, long‐term health monitoring and treatment, creating scalable solutions for both diagnostic and therapeutic applications in wearable health devices (Figure 8f).^[^ ^139^ ^]^ These advancements highlight the transformative potential of photonic NMs in wearable health devices, where seamless and efficient data communication is critical for non‐invasive, long‐term health monitoring and treatment.

## 5 Applications of Wearable Health

Wearable health technology offers innovative solutions for precise diagnostics, real‐time health monitoring, and therapeutic functions by seamlessly interfacing with the body. These advancements position wearable devices as a cornerstone of personalized medicine and preventive health.^[^ ^2^, ^34^, ^140^ ^]^ Among the various formats, skin patches and contact lenses stand out for their accessibility, versatility, and ability to integrate seamlessly with the body. Skin patches provide an adaptable platform for continuous monitoring of physiological parameters directly from the skin, while contact lenses facilitate direct interaction with the eye's tear fluid and pressure systems, enabling precise ocular diagnostics and treatments.^[^ ^2^, ^5^, ^16^, ^140^, ^141^ ^]^ The non‐invasive nature of these devices, combined with their capability to provide continuous data, enhances user compliance and supports a broad range of applications across various health conditions. Skin patches are particularly effective for applications such as sweat analysis, photobiomodulation, and photoplethysmography (PPG), where they enable diagnostic, therapeutic, and monitoring functions. These devices analyze biomarkers from sweat, utilize light for cellular stimulation and wound healing, and monitor blood flow for cardiovascular health. Contact lenses, on the other hand, are valuable for analyzing tear fluid, delivering drugs in a controlled manner, and measuring intraocular pressure, making them essential for diagnosing and treating ocular diseases. By integrating optical sensing technologies, drug delivery systems, and microstructured sensors for pressure and fluid measurements, these devices advance continuous health status monitoring, early diagnosis, and personalized treatments. These wearable health technologies represent a significant step forward in tailored health management, contributing to early diagnosis and prevention of various diseases while advancing the field of personalized medicine. Furthermore, the integration of these components facilitates the development of closed‐loop systems, which enable real‐time monitoring and automated feedback for adaptive and precise treatment. Expanding on this concept, such systems exemplify the potential for seamless interaction between sensing and therapeutic functions, offering detailed examples like insulin pumps for diabetes management or wearable devices for controlled drug release based on physiological feedback.

### 5.1 Skin Patch Applications

Wearable sensor technology represents a burgeoning commercial opportunity in the fields of non‐invasive biomedical research on diagnostic, therapeutic, and monitoring. The most advanced versions of wearable sensors demand direct and seamless contact with human skin to reliably record critical physiological and electrophysiological signals.^[^ ^34^, ^51^, ^140^, ^142^ ^]^ Recent advances in materials science, mechanics, and electronics have established a foundation for stretchable, flexible, and ultra‐thin electronic devices capable of conforming to the complex, textured surfaces of human skin, even under dynamic skin deformation.^[^ ^143^ ^]^ The devices discussed highlight the most advanced technologies designed for seamless integration with human skin, offering practical solutions for continuous and precise health monitoring in everyday settings. **Table** **3** presents a summarized overview of these devices in terms of their clinical implementations, biomarkers/biosignals, working principles, and representative materials.

**Table 3:** A summarized overview of the skin patch monitoring systems, highlighting their clinical implementations, targeted biomarkers/biosignals, working principles, and representative materials utilized for diagnostic, therapeutic, and monitoring applications.

	Clinical applications	Biomarkers/biosignals	Working principles	Representative materials	Refs.
Diagnostic	Sweat analysis	Lactate, glucose, chloride, pH, urea, ammonia, lidocaine, methotrexate, electrolytes	Measurement of shifts in optical properties of sweat biomarkers	Au NPs, Core–shell, Graphene, PNs	[145, 146, 147, 148, 149, 150]
Therapeutic	Photobiomodulation	ROS, Adenosine triphosphate (ATP), Nitric oxide (NO), Blood flow, oxygenation, collagen synthesis, cell proliferation	Modulation of cellular activity through specific wavelengths of light absorbed by chromophores in tissues, triggering biochemical changes	Au NPs, Metal nanoshell, QDs, CNTs, PCs	[2, 15, 69, 156, 157, 158, 159, 160]
Monitoring	Photoplethysmography	Heart rate (HR), Heart rate variability (HRV), Saturation of peripheral oxygen (SpO_2_), respiration rate, Pulse transit time (PTT)	Detection of blood volume changes in microvascular tissue, where variations in intensity correspond to the pulsatile nature of blood flow	QDs, Perovskite	[124, 164, 165, 166]

#### 5.1.1 Sweat Analysis

Sweat sensing is crucial for non‐invasive health monitoring, offering valuable insights into biomarkers such as lactate, glucose, chloride, pH, and electrolytes.^[^ ^144^ ^]^ Unlike invasive methods like blood sampling, sweat can be painlessly collected from the skin, making it ideal for continuous monitoring. Biomarkers in sweat provide critical information about physical stress, hydration levels, and metabolic states. For instance, lactate levels indicate transitions from aerobic to anaerobic states, while glucose levels aid in diabetes management.^[^ ^144^, ^145^ ^]^ Despite its potential, current sweat monitoring methods face significant challenges. Traditional approaches involve attaching filter paper or gauze to the skin to absorb sweat, followed by laboratory analysis using techniques like liquid chromatography or mass spectrometry. While effective, these methods are impractical for prompt detection and prone to inaccuracies due to sample degradation, contamination, and loss during collection, storage, and transport. Moreover, variations in sweat secretion rates and environmental factors complicate data interpretation, limiting their utility in dynamic, on‐body scenarios.^[^ ^13^, ^34^, ^142^, ^146^ ^]^ Photonic NMs, including Au NPs, core–shell nanostructures, graphene‐based materials, and PNs, provide innovative solutions to these limitations.^[^ ^145^, ^146^, ^147^, ^148^, ^149^, ^150^ ^]^ Plasmonic NMs, such as Au NPs and Au nanostructures, enhance detection sensitivity using SERS, enabling precise molecular fingerprinting. Core–shell designs improve stability, protecting active components, while graphene offers flexibility, robustness, and compatibility for multiplexed sensing. These materials also reduce signal drift and enhance measurement accuracy by minimizing environmental interference.

**Figure 9:**
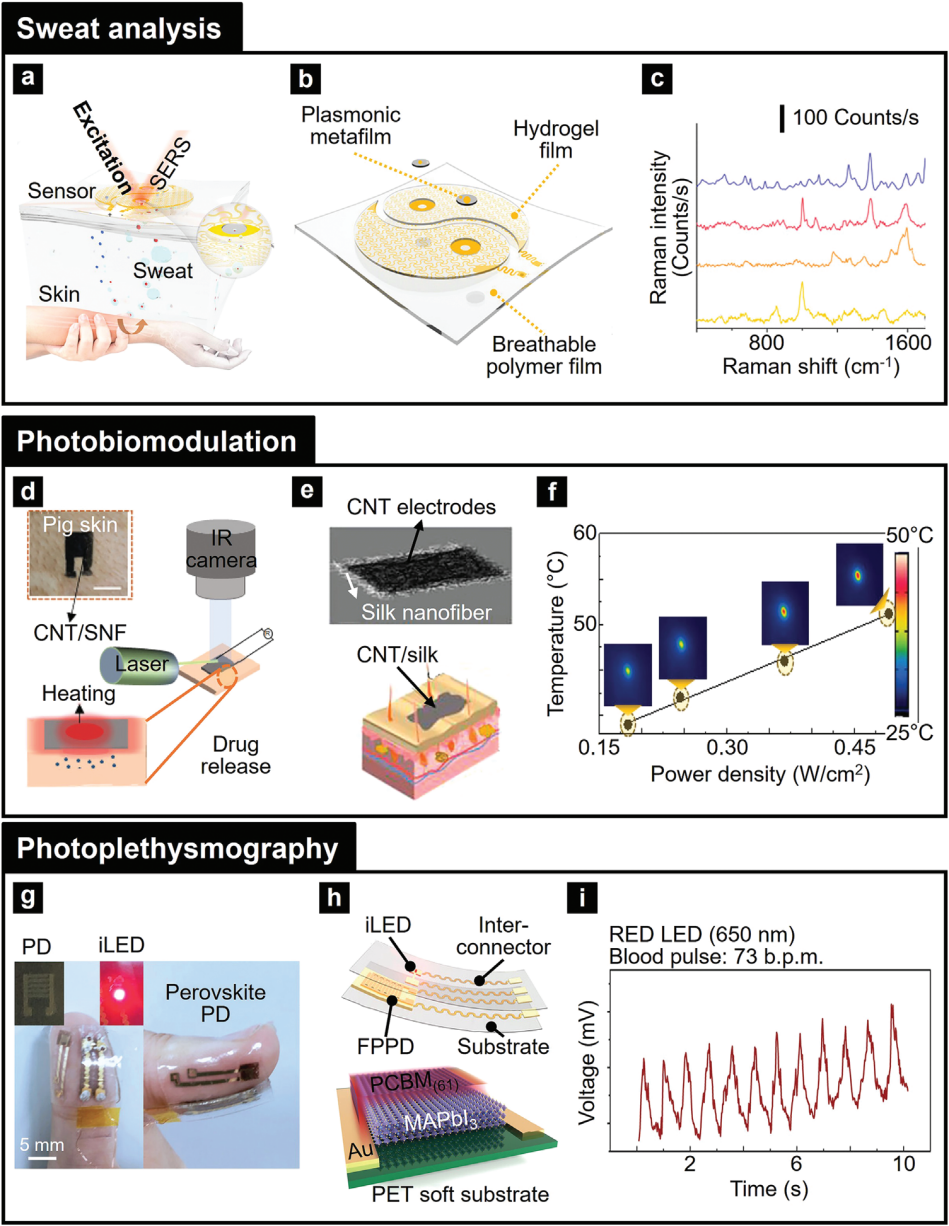
Skin patch applications. a) Schematic illustration of the working principle and design of the device. b) The components of sweat extraction and surface‐enhanced Raman scattering (SERS) sensing modules. c) SERS spectra of the human sweat samples (yellow line) compared to various drugs: 10–5 m methotrexate (orange line), 10–3 m cocaine (red line), 0.2 m lidocaine (blue line). Reproduced with permission.^[^ ^150c^ ^]^ Copyright 2021, American Association for the Advancement of Science. d) Schematic illustration of an optically induced heating element under laser illumination. e) Schematic of an electronic tattoo (E‐tattoo) and demonstration of mechanical stability and conformal adhesion of carbon nanotube (CNT)/silk nanofiber (SNF) E‐tattoo stickers on the skin. f) Heater response as a function of incident power density, illustrated with infrared (IR) images. Reproduced with permission.^[^ ^159a^ ^]^ Copyright 2021, Wiley‐VCH. g) Physical schematic of the flexible photoplethysmography (PPG) signal sensor (left inset: flexible perovskite photodetector; right inset: red LED used as a light source). h) Detailed structural diagram of the flexible PPG signal sensor (iLED: inorganic LED, FPPD: flexible perovskite photodetector (MAPbI3–PCBM)). i) PPG signal recorded within 10 s at wavelength: 650 nm. Reproduced with permission.^[^ ^164^ ^]^ Copyright 2023, Wiley‐VCH.

Integrating photonic NMs into wearable sensors enables proactive sweat analysis with high sensitivity and specificity. These advanced devices overcome the drawbacks of traditional methods, providing reliable, on‐site monitoring for health assessment. The combination of surface‐interaction‐based collection and advanced NM functionality positions sweat sensors as key tools for personalized medicine and disease management. **Figure** **9**a illustrates the most advanced concept in the domain of wearable photonic NMs, integrating a flexible and stretchable plasmonic metasurface directly mounted on human skin, where the embedded SERS‐active Ag nanocube (NCs) arrays provide molecular fingerprinting and analysis.^[^ ^150c^ ^]^ This plasmonic metafilm, shown in high‐resolution in Figure 9b, is composed of a densely packed, highly ordered monolayer of Ag NCs with nanoscale gaps (≈1 nm), ensuring the creation of strong electromagnetic hotspots critical for high‐sensitivity detection. The wearable sensor extracts sweat non‐invasively using iontophoresis induced by a hydrogel containing 10% acetylcholine chloride. Sweat analytes are transported to the plasmonic metasurface, where molecular fingerprints are captured via SERS. This platform demonstrated instant detection of various biomarkers and drugs in sweat, including nicotine and chemotherapy agents like mitoxantrone.^[^ ^151^ ^]^ For instance, nicotine levels were monitored dynamically when a 10 mg nicotine patch was applied, with the sensor recording precise temporal and spatial variations in nicotine concentration.^[^ ^152^ ^]^ These measurements, achieved at a sensitivity of 0.01 nM, underscore the sensor's ability to analyze trace molecular concentrations from minimal sweat volumes, as low as <0.5 µL. Additionally, the plasmonic metasurface identified pH variations and drug profiles with high specificity, using the characteristic SERS spectrum of each molecule (Figure 9c). The sensor array design allowed the simultaneous detection of multiple analytes, including SERS‐inactive targets, by incorporating probe molecules such as 4‐mercaptopyridine (4‐MTP) for pH monitoring. The integration of multiple sensors on a lightweight, breathable, and stretchable substrate ensured accurate sweat analysis even under dynamic mechanical strains of up to 30% without signal degradation.

Future development of this sensor will focus on enhancing portability by integrating miniaturized Raman spectrometers or smartphone‐compatible systems for on‐site signal readout.^[^ ^144^, ^153^ ^]^ Additionally, a microchannel system could be incorporated to control sweat collection and improve analyte quantification accuracy. Expanding the base material of the sensor to more stable metals, such as Au or Pt, would address potential concerns about Ag corrosion and environmental stability.^[^ ^154^ ^]^ Further research will also explore correlating sweat biomarkers with blood or interstitial fluid levels to enable broader health monitoring applications, including tailored drug therapy and chronic disease management.

#### 5.1.2 Photobiomodulation

Photobiomodulation is a non‐invasive therapeutic technique that uses light to modulate cellular activity, offering applications in various medical fields such as wound healing and nerve regeneration. However, conventional photobiomodulation technologies face challenges including limited tissue penetration of light, inefficient energy delivery, and significant losses due to scattering and absorption, which hinder deep‐tissue treatment and precision medicine.^[^ ^5^, ^141^, ^155^ ^]^ To address these limitations, photonic NMs such as Au NPs, metal nanoshells, QDs, CNTs, and PCs have been integrated into photobiomodulation systems.^[^ ^69^, ^156^, ^157^, ^158^, ^159^, ^160^ ^]^ Au NPs amplify light energy through PR, enhancing energy delivery to target tissues. Metal nanoshells efficiently utilize NIR light to penetrate deeper into tissues. QDs enable precise control over light wavelengths to selectively stimulate specific cells or tissues. CNTs absorb a broad spectrum of light, delivering energy efficiently while offering exceptional flexibility for application in dynamic environments. PCs minimize light scattering, optimize energy transfer, and facilitate real‐time treatment monitoring. Collectively, these NMs significantly improve the capabilities of photobiomodulation for deep‐tissue treatment and precision, unlocking its potential in managing chronic diseases, regenerative medicine, and neurorehabilitation. The incorporation of photonic NMs into photobiomodulation technologies paves the way for groundbreaking advancements, expanding the scope and efficacy of this therapeutic modality in modern medicine.

Figure 9d demonstrates one of the most promising concepts in wearable photonic systems: a flexible and ultra‐thin CNT‐functionalized silk nanofiber (SNF) electronic tattoo (E‐tattoo).^[^ ^159a^ ^]^ This innovative platform (Figure 9e) can be seamlessly mounted on human skin, offering capabilities for photobiomodulation and diagnostics. The CNT‐based system utilizes its high optical absorption and thermal conductivity to efficiently convert light into heat, enabling targeted therapeutic and diagnostic functions. The E‐tattoo exhibits exceptional potential in photobiomodulation, harnessing the optical absorption of CNTs to generate localized heating upon light stimulation. When illuminated with a 532 nm green laser at varying power densities, the E‐tattoo achieved precise temperature control, with thermal uniformity across its surface. At a power density of 0.48 W cm^‒2^, the system reliably reached 50 °C, suitable for therapeutic applications (Figure 9f). The photothermal conversion efficiency of the CNT layer enables non‐invasive, wireless heating, distinguishing it from conventional electrically powered systems.^[^ ^161^ ^]^ To validate its photobiomodulation capabilities, the E‐tattoo was tested on pig skin as a model system. The heating performance was paired with Rhodamine B‐loaded silk membranes to explore drug delivery enhancement. Optical stimulation drove drug penetration into the skin at depths exceeding 500 µm, significantly outperforming electrically heated samples (≈270 µm). This highlights the ability of the E‐tattoo to enhance cellular and tissue interaction via heat‐mediated therapy while minimizing invasive procedures. The system also allows dual functionality as a heater and temperature sensor. The embedded CNT/SNF structure provides prompt temperature feedback, ensuring precise thermal control during photobiomodulation. The temperature coefficient of resistance (TCR) for the CNT layer was measured at 5.2 × 10^−3^ °C^−1^, enabling dynamic response adjustments to maintain safe and effective treatment conditions.^[^ ^162^ ^]^

Future developments aim to enhance the portability and versatility of the E‐tattoo system by integrating wireless, LED‐based illumination systems to replace lasers, enabling compact, wearable, and safe light sources for photobiomodulation. Additionally, exploring therapeutic wavelengths beyond the current spectrum could broaden applications in tissue repair, inflammation reduction, and pain relief. To further improve the efficiency of the system, optimizing the CNT dispersion process will enhance optical absorption while reducing power requirements, making the device more energy‐efficient.

#### 5.1.3 Photoplethysmography

PPG is a non‐invasive optical technique for measuring blood flow and cardiovascular status, providing critical physiological data such as heart rate, SpO_2_, and blood pressure. It plays a significant role in health monitoring and disease management due to its simplicity, low cost, and ease of integration into wearable devices, making it a widely used tool for cardiovascular health tracking and patient care. However, conventional PPG technologies face limitations in measurement accuracy and efficiency.^[^ ^163^ ^]^ Traditional PPG relies on LEDs and photodetectors to measure light absorbed and reflected by blood, but challenges such as signal degradation, limited light penetration depth, and fixed wavelength constraints hinder precise data collection. Physiological and environmental factors, including body motion, skin thickness, and pigmentation, introduce noise, compromising reliability, especially in users with dark skin, thick tissue, or during physical activity.^[^ ^163^, ^164^ ^]^

Photonic NMs have emerged as a solution to overcome these challenges, significantly advancing PPG technology. QDs and perovskites enhance PPG accuracy and efficiency through their unique optical properties and design flexibility.^[^ ^124^, ^164^, ^165^, ^166^ ^]^ QDs can be tailored to emit specific wavelengths, enabling precise measurement of biomarkers such as SpO_2_, while their high PL efficiency improves the signal‐to‐noise ratio (SNR). They exhibit stable performance in biological environments, making them ideal for long‐term and repetitive measurements. Perovskites offer superior light absorption and spectral selectivity, effectively delivering light to deeper tissues even with thin structures, and addressing the penetration limitations of conventional PPG. Their cost‐effective production of high‐performance light sources enhances the economic feasibility of wearable devices, while their excellent charge transport properties improve signal quality and physiological data accuracy.

Integrating photonic NMs allows PPG technology to achieve accurate blood flow measurements regardless of tissue depth, effectively mitigating signal distortion caused by environmental and physiological factors.^[^ ^163^, ^167^ ^]^ These materials ensure reliable data during physical activity and for users with darker skin or thicker tissues. Furthermore, the ease of incorporating QDs and perovskites into wearable devices positions PPG as a pivotal technology for personalized health monitoring and disease prevention. The introduction of photonic NMs not only overcomes the limitations of conventional PPG but also enhances its precision and reliability, unlocking new possibilities for customized health and establishing a new benchmark for health monitoring technologies.

Figure 9g illustrates one of the promising advancements in wearable optoelectronics: a miniaturized, flexible, and stretchable PPG sensor that integrates a perovskite‐based photodetector (MAPbI_3_–PCBM) and an inorganic red LED.^[^ ^164^ ^]^ This innovative system can be directly mounted on human skin, enabling continuous monitoring of cardiovascular signals and limb health. The embedded photonic NMs, specifically perovskite QDs, provide high sensitivity, mechanical flexibility, and adaptability to irregular surfaces such as finger joints. The perovskite photodetector, built using a solution‐processed MAPbI_3_–Phenyl‐C_61_‐butyric acid methyl ester (PCBM) heterojunction, leverages the superior optical absorption and high carrier mobility of perovskite QDs (Figure 9h).^[^ ^168^ ^]^ It achieves a responsivity of 120.7 mA W^‒1^ at 635 nm under faint lighting conditions (0.25 mW cm^‒2^) and demonstrates a photocurrent of 8.33 µA at a power density of 64.4 mW cm^‒2^. The introduction of PCBM as an electron transport layer enhances crystallinity and reduces non‐radiative recombination, ensuring high performance in low‐light conditions. When used in the PPG sensor, the photodetector captures subtle variations in reflected light caused by blood vessel pulsations and tissue swelling. The device monitors changes in signal amplitude to detect finger swelling, achieving reductions of 68.7% in DC signals and 30.4% in AC signals under severe swelling conditions. The photodetector's rapid response time of 12 ms (rise) and 9 ms (fall) ensures accurate data acquisition, making it suitable for monitoring heart rate and blood oxygen levels (Figure 9i). The mechanical adaptability of the sensor is enhanced by a 3D wrinkled‐serpentine interconnection structure, which provides robust performance under bending and stretching. The photodetector retains 93.2% efficiency even at a bending angle of 60°, and the interconnection design supports tensile strains of up to 120%, ensuring durability for wearable applications on curved or flexible surfaces like finger joints.

Future developments will focus on miniaturizing the sensor for seamless integration into everyday health‐monitoring devices while improving the stability of perovskite materials against environmental factors such as moisture and oxygen. Expanding the spectral range of the system to include wavelengths for therapeutic applications, such as wound healing or inflammation reduction, could further enhance its versatility. Additionally, incorporating wireless communication capabilities and exploring biodegradable substrates may advance its usability and environmental sustainability.

### 5.2 Contact Lens Applications

Contact lenses hold significant promise as eye‐mountable biomedical platforms due to their simple and noninvasive access to the body's internal chemistry (tear film) and the outermost layer of the eye (cornea).^[^ ^169^ ^]^ However, current contact lenses are primarily designed for vision correction or passive ocular drug delivery. Recent advancements have enabled the development of contact lens‐based sensors for noninvasive and continuous monitoring of metabolites in tear film, such as glucose and lactate.^[^ ^80^, ^170^ ^]^ These platforms also show potential for early diagnosis, precise therapy, and real‐time monitoring of ocular diseases, including glaucoma, cataracts, and retinal disorders.^[^ ^170^, ^171^ ^]^ The following sections highlight key examples of eye‐mountable health monitoring devices, categorized by their clinical applications: i) tear fluid analysis, ii) drug release systems, and iii) pressure measurement. **Table** **4** provides a concise summary of these devices, highlighting their clinical applications, targeted biomarkers or biosignals, operational principles, and representative materials.

**Table 4:** Overview of the contact lens‐based monitoring systems, summarizing their clinical implementations, targeted biomarkers/biosignals, working principles, and representative materials used for diagnostic, therapeutic, and monitoring applications.

	Clinical applications	Biomarkers/biosignals	Working principles	Representative materials	Refs.
Diagnostic	Tear fluid analysis	Lactate, glucose, proteins, pH, cytokines, electrolytes	Detection of biomarker‐induced changes in optical properties	Au NPs, QDs, 2D TMDCs, Graphene, CNTs, nanograting	[38, 51, 65, 80, 82, 170, 172, 173, 174, 175, 176]
Therapeutic	Drug release systems	Drug concentration, release rate profiles, tear pH, temperature, osmolarity	Controlled release of drugs from contact lens materials through diffusion, degradation, or stimulus‐responsive mechanisms	Au NPs, GQDs, PCs	[32, 38, 64, 82, 172, 180, 181, 182]
Monitoring	Pressure measurement	IOP, corneal deformation pattern, mechanical stress on the lens	Measurement of corneal deformation‐induced changes, such as capacitance, resistance, or optical signals	Au, Ag, PCs	[32, 70, 80, 172, 189]

#### 5.2.1 Tear Fluid Analysis

Tear analysis using contact lenses offers a non‐invasive method for live‐streamed monitoring of health conditions, playing a pivotal role in diagnosing and managing diseases such as diabetes, ocular disorders, and dehydration. Traditional approaches have been limited by their invasive nature, inconvenience, and inability to provide immediate response evaluation. However, advances in photonic NMs have effectively addressed these challenges.^[^ ^32^, ^172^ ^]^ Photonic NMs enable the creation of highly sensitive sensors for the accurate detection of biomarkers in tears. Au NPs utilize SPR to amplify signals and can be functionalized to selectively target specific biomarkers, enhancing analytical precision.^[^ ^173^ ^]^ QDs offer unique photoluminescent properties, emitting light at specific wavelengths for multiplexed biomarker detection.^[^ ^38^, ^82^, ^172^, ^174^ ^]^ Their high PL efficiency significantly improves signal quality, enabling precise and reliable monitoring. 2D TMDCs, with their ultra‐thin structure and exceptional sensitivity, integrate seamlessly with contact lenses without obstructing vision, ensuring accurate data collection.^[^ ^65^, ^172^ ^]^ Graphene, known for its high electrical conductivity and large surface area, amplifies electrochemical signals and exhibits excellent flexibility and durability, making it ideal for integration into lenses.^[^ ^170^, ^175^ ^]^ CNTs absorb light across a broad spectrum, delivering high sensitivity and durability for extended wear.^[^ ^51^, ^80^, ^172^ ^]^ Nanogratings, by manipulating light reflection and diffraction, improve sensor sensitivity and selectivity, supporting comprehensive tear analysis across multiple wavelengths.^[^ ^80^, ^174^, ^176^ ^]^ Contact lens‐based tear analysis overcomes the limitations of conventional methods through the use of photonic NMs, enabling real‐time monitoring and personalized health management. This technology represents a transformative leap in health, establishing itself as a cornerstone of next‐generation medical solutions while unlocking new possibilities in diagnostics and disease management.

**Figure 10:**
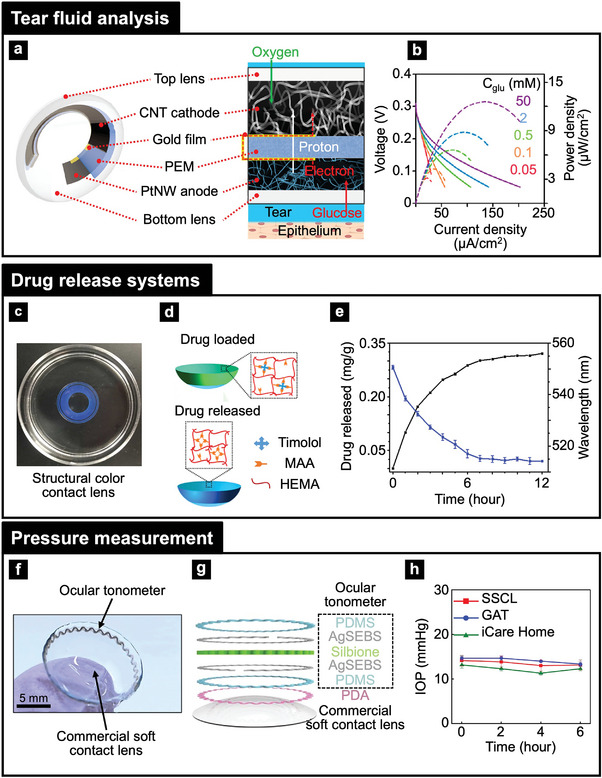
Contact lens applications. a) Schematic illustration of the smart contact lens structure and the operation of the embedded glucose fuel cells. b) Polarization curves of our glucose fuel cells integrated into hydrogel lenses, measured under glucose concentration ranging from 0.05 to 50 mm. Reproduced with permission.^[^ ^80f^ ^]^ Copyright 2022, American Chemical Society. c) Digital photograph showcasing a structural color contact lens. d) Schematic of the drug loading and releasing mechanism. MAA: methacrylic acid. HEMA: hydroxyethyl methacrylate. e) Accumulative drug release profile and corresponding reflection peak shift during release in artificial tear fluid (ATF). Reproduced with permission.^[^^182b]^ Copyright 2018, American Chemical Society. f) Photograph of the smart soft contact lenses (SSCL). g) Layered schematic diagram illustrating the structural components of the SSCL. h) Time‐dependent variation in the ambulatory intraocular pressure (IOP) measured over 6 h using the SSCL (red line), compared with Goldmann applanation tonometry (GAT) (blue line), and iCare Home (green line) measurements (n = 3). Reproduced with permission.^[^^189]^ Copyright 2022, Springer Nature.

**Figure** **10**a highlights one of the most promising concepts in the realm of miniaturized, flexible, and wearable devices: self‐powered smart contact lenses that incorporate advanced photonic NMs for continuous glucose sensing and visual feedback.^[^ ^80f^ ^]^ These lenses integrate a glucose fuel cell, built using NMs such as CNTs, Pt NWs, and Au films, alongside embedded PC arrays for direct glucose‐level visualization. The flexible design allows the lenses to conform seamlessly to the eye while ensuring long‐term comfort, safety, and durability. The glucose fuel cells employ a layered structure featuring Pt NWs as the anode, a proton exchange membrane (PEM), and a CNT‐based porous cathode for efficient fuel oxidation and oxygen reduction. The cathodes, fabricated using stencil‐coating techniques, exhibit high porosity and enhanced mass transfer, improving glucose diffusion and reaction rates.^[^ ^177^ ^]^ This configuration achieves power densities of 4.4 µW cm^‒2^ at 0.05 mm glucose (normal tear glucose concentration) and 8.8 µW cm^‒2^ at 2 mm (diabetic levels), surpassing most existing abiotic fuel cells (Figure 10b). In addition to power generation, the contact lenses feature PC arrays embedded in electroactive hydrogels. These arrays provide structural color changes in response to glucose concentrations, enabling visible differentiation between normal and diabetic glucose levels. For instance, at glucose levels above 1 mm, the PC shifts from infrared to visible wavelengths, producing discernible red tones for user‐friendly, naked‐eye detection. The NM‐based structure demonstrates remarkable durability and mechanical resilience. The lenses maintain functionality after being bent in half over 100 cycles and continue to operate reliably after two weeks of storage in aqueous solutions. Moreover, the oxygen permeability of the hydrogel lenses ensures sufficient corneal oxygenation during wear, preventing discomfort or eye damage.

Looking forward, future advancements will aim to miniaturize the photonic components and incorporate wireless communication modules for continuous, seamless glucose monitoring and data transmission. Expanding the functionality of PCs to detect additional analytes, such as lactate or ascorbate, could transform the lenses into a multi‐analyte diagnostic platform. Furthermore, adopting alternative NMs, such as graphene or TMDCs, could improve device efficiency, conductivity, and long‐term biocompatibility.

#### 5.2.2 Drug Release Systems

Contact lens‐based drug release systems offer an innovative approach for treating ocular diseases, overcoming limitations of conventional eye drops and ointments, which often suffer from low drug delivery efficiency due to rapid washout by tears and limited tissue absorption. These advanced systems enable sustained and controlled drug release, maximizing therapeutic efficacy and improving patient convenience. However, traditional systems face challenges such as uneven drug release, insufficient control, and issues with drug stability, necessitating further refinement.^[^ ^80^, ^172^, ^178^ ^]^ Conventional approaches often release drugs too rapidly in the initial phase, failing to sustain therapeutic effects over time. Moreover, they lack precise control over drug release rates and quantities, limiting their adaptability to specific disease requirements. Drug degradation and reduced activity over time further hinder their efficacy.^[^ ^176^, ^179^ ^]^ To address these issues, photonic NMs such as Au NPs, GQDs, and PCs have been incorporated, revolutionizing the sensitivity and control of drug delivery systems. Au NPs leverage photothermal effects to generate heat upon light interaction, enabling precise control of drug release.^[^ ^64^, ^180^ ^]^ Their exceptional chemical stability ensures prolonged maintenance of drug efficacy. GQDs respond to specific wavelengths of light to trigger drug release, offering low toxicity and excellent biocompatibility, making them ideal for ocular applications.^[^ ^38^, ^82^, ^181^ ^]^ PCs manipulate light reflection and refraction to fine‐tune drug release rates, with customizable structures designed to optimize drug delivery for specific conditions.^[^ ^32^, ^172^, ^182^ ^]^ The incorporation of these photonic NMs allows contact lens‐based drug delivery systems to address inefficiencies and lack of control in traditional methods. Au NPs enable precision release control, GQDs enhance delivery efficiency with high sensitivity and biocompatibility, and PCs provide tailored release profiles. In conclusion, photonic NM‐enhanced contact lens‐based drug delivery systems pave the way for next‐generation ocular treatments, offering non‐invasive and highly effective therapeutic solutions. This technology significantly improves patient convenience and therapeutic outcomes while positioning itself as a key foundation of personalized health. The advancement of precision and sustainability in drug delivery paves the way for treating ocular diseases with unprecedented efficacy and adaptability.

Figure 10c illustrates one of the most promising advancements in the field of nanostructured, functional devices: molecularly imprinted structural color contact lenses that integrate Au NPs, GQDs, and PCs.^[^ ^182b^ ^]^ These lenses serve as a dual‐function platform for sustained ophthalmic drug delivery and real‐time visual monitoring of drug release via structural color changes. The embedded photonic NMs enable precise drug loading, controlled release, and colorimetric feedback, marking a significant leap in non‐invasive therapeutic technologies. The structural color of the contact lenses arises from their inverse opal PC architecture, which is fabricated using a template of monodispersed silica NPs (200 nm). This highly ordered, interconnected porous structure ensures efficient molecular imprinting, significantly enhancing drug‐binding capacity.^[^ ^183^ ^]^ The embedded nanostructure reflects light at specific wavelengths based on the Bragg diffraction peak, enabling visible color changes during drug loading and release (Figure 10d). During drug loading at pH 6.5, the lenses exhibit a redshift of 12.4 nm in the reflection peak, attributed to lattice expansion as timolol molecules bind within the imprinted cavities.^[^ ^184^ ^]^ In contrast, drug release in artificial tear fluid (pH 8.0) induces a 36.4 nm blueshift, caused by lattice contraction as the drug molecules diffuse out.^[^ ^185^ ^]^ These optical changes are directly visible to the naked eye, allowing users to monitor the drug release process in real‐time without external instrumentation. The molecular imprinting process, facilitated by the photonic NMs, enables the lenses to achieve a drug‐loading capacity of 0.3484 mg g^‒1^, more than double that of non‐imprinted lenses (Figure 10e). This capacity is further enhanced by the high surface area and molecular selectivity of the nanostructured hydrogel matrix. Additionally, the lenses demonstrate excellent reusability, maintaining consistent loading and release performance over five cycles, aligning with daily wear and reload schedules.

Future advancements aim to enhance the optical resolution and sensitivity of structural color changes by incorporating Au NPs and GQDs with tunable optical properties. Expanding the platform for multi‐drug delivery and integrating sensors for detecting multiple analytes, such as pH or ion concentrations, will broaden the diagnostic capabilities.

#### 5.2.3 Pressure Measurement

Contact lens‐based IOP measurement technology plays a critical role in the early diagnosis and management of glaucoma by providing live‐streamed monitoring of IOP. Elevated IOP can cause optic nerve damage and, if left untreated, lead to irreversible blindness. Therefore, precise and effective pressure monitoring is essential. However, traditional IOP measurement methods face limitations in precision and convenience, necessitating the development of more innovative approaches.^[^ ^32^, ^172^, ^175^, ^186^ ^]^ Traditional methods typically involve sporadic measurements performed in clinical settings, failing to capture daily IOP fluctuations. Many methods require corneal contact or anesthesia, causing discomfort and inconvenience for patients. Measurement accuracy can also vary due to environmental factors or operator skill.^[^ ^32^, ^186^, ^187^ ^]^ To address these challenges, photonic NM‐enhanced contact lens‐based pressure measurement systems have emerged, leveraging materials such as Au, Ag, and PCs to deliver high sensitivity and accuracy. Au interacts with light to amplify optical signals in response to pressure changes, enabling precise IOP detection. Their chemical stability and biocompatibility ensure safe and reliable measurements over extended wear periods.^[^ ^188^ ^]^ Ag NMs exhibit rapid optical responsiveness and high reflectivity, making them highly sensitive to pressure variations. They amplify weak optical signals, ensuring fine‐tuned sensitivity even under challenging conditions.^[^ ^32^, ^172^, ^189^ ^]^ PCs use structural color changes induced by pressure variations to visually represent or sensor‐detect IOP changes. Their customizable designs allow for precise pressure range optimization, supporting the management of various ocular conditions.^[^ ^70^, ^80^ ^]^ In conclusion, photonic NM‐enhanced contact lens‐based pressure measurement technology addresses the shortcomings of traditional methods, providing a revolutionary solution for IOP monitoring. This innovation enables early diagnosis and continuous management of glaucoma and other ocular diseases, simultaneously enhancing patient convenience and diagnostic accuracy. As a cornerstone of next‐generation health technologies, it opens new possibilities for precision and sustainability in ocular disease management.

Figure 10f highlights one of the most promising innovations in wearable ophthalmic devices: smart soft contact lenses (SSCL) equipped with advanced photonic NMs such as Au NPs, Ag flakes, and PCs. These lenses integrate a stretchable, serpentine‐shaped resistor‐inductor‐capacitor (RLC) resonant circuit directly onto commercial soft contact lenses, enabling real‐time, non‐invasive, 24 h monitoring of IOP while maintaining comfort, transparency, and biocompatibility essential for long‐term wear (Figure 10g).^[^ ^189^ ^]^ The SSCL leverages photonic NMs to achieve precise and wireless IOP monitoring. The embedded RLC circuit, incorporating Ag flakes within a flexible elastomer, detects mechanical deformations caused by changes in IOP. These deformations induce shifts of inductance and capacitance in the circuit, leading to resonance frequency changes that are wirelessly transmitted to a nearby reader coil embedded in accessories like eyeglasses or sleep masks.^[^ ^190^ ^]^ With a sensitivity of 0.27 MHz mmHg^‒1^, the SSCL surpasses current state‐of‐the‐art wearable tonometers, reliably measuring IOP across a range of 0–40 mmHg (Figure 10h).^[^ ^170^, ^186^, ^191^ ^]^ The integration of PCs enhances the lens's functionality by providing visible structural color changes, which could enable intuitive visual feedback for users in future designs. The SSCL demonstrates exceptional durability and stability, with a PDMS encapsulation layer that protects against environmental factors such as moisture, disinfectants, and extreme temperatures ranging from −4 to 75 °C. The lens maintains its performance even after 10 000 mechanical cycles of stretching and rubbing, ensuring reliability during daily wear. In vivo experiments conducted in rabbit and human eyes reveal that the SSCL conforms seamlessly to diverse corneal shapes and thicknesses, providing accurate and repeatable measurements without compromising wearer comfort. Histological analysis confirms minimal to no inflammation after extended use, and participants reported comfort levels comparable to those of standard contact lenses.

Future advancements aim to miniaturize wireless communication modules and incorporate multi‐analyte sensing capabilities for detecting additional biomarkers like tear glucose or oxygen levels. The adoption of next‐generation photonic NMs, such as plasmonic Au NPs and optimized PC structures, could further improve sensitivity and expand the device's utility to other ocular diseases, including macular degeneration and cataracts. Additionally, refining the fabrication process for scalable and cost‐effective production will be essential for widespread clinical adoption.

## 6 Future Perspectives in Photonic Applications

Despite significant advancements in designing photonic NMs, their translation into wearable device applications remains limited, particularly in realizing their full potential for sensing and therapeutic functionalities.^[^ ^5^, ^11^, ^15^, ^192^ ^]^ Machine learning (ML)‐assisted design and systematic integration hold promise for overcoming these limitations, enabling durable, high‐performance wearable devices that address the specific needs of sensitive environments such as the skin and eye.^[^ ^193^ ^]^

Photonic design challenges are often tackled by solving Maxwell's equations through numerical techniques like the finite element method (FEM) and finite‐difference time‐domain (FDTD) method. These techniques are commonly used for inverse design tasks, where the goal is to determine the optimal structure to achieve specific optical responses and functionalities. The standard process typically involves conducting full‐wave simulations of an initial design informed by empirical knowledge, followed by iterative adjustments to geometric and material parameters to fulfill predefined requirements. However, this trial‐and‐error approach can be highly time‐intensive, even for experienced researchers. While these methods are well‐established and effective, they may constrain the exploration of novel designs, particularly for advanced applications where traditional approaches fall short.

Machine learning, in particular deep learning, can offer a transformative approach to photonic design for wearable health devices, leveraging their ability to establish complex relationships between inputs, such as geometric or material parameters, and outputs of optical responses. Machine learning enables a more efficient workflow, eliminating the reliance on computationally intensive simulations and optimization routines. Deep learning can be employed in two key functions for photonic design: forward prediction and inverse design. Forward prediction models estimate optical responses based on given geometric or material parameters, providing a faster alternative to traditional full‐wave simulations. In contrast, inverse design models focus on identifying the optimal structure for achieving desired optical responses, addressing one of the most critical and challenging aspects of the design process. One of the primary advantages of deep learning would be their speed, significantly outpacing traditional simulation methods, such as FEM or FDTD. Deep learning excels in uncovering nonintuitive and nonunique relationships between physical structures and optical properties, offering researchers insights into entirely new classes of designs that may not be apparent through conventional approaches.

Addressing the design challenges of photonic‐based wearable health devices with deep learning relies on a data‐driven methodology, necessitating extensive training datasets that pair geometric or material parameters with their corresponding optical responses. Once a deep learning model demonstrates strong performance on a training dataset, it is evaluated using a separate test dataset or applied to real‐world problems. Importantly, the training and test datasets must operate within the same design framework but consist of entirely distinct data. For inverse design tasks, the roles of inputs and outputs are reversed, and a similar network structure can often be utilized. However, certain problems may require more advanced methods and algorithms to address their complexity effectively.

Deep learning such as neural networks (NNs), convolutional neural networks (CNNs), reinforcement learning (RL), and generative adversarial networks (GANs) enables rapid optimization, inverse design, and the exploration of complex design spaces. Despite notable progress in optimizing photonic NMs for sensing, energy systems, and quantum photonics, their application in wearable devices remains underdeveloped. AI‐assisted design can address critical barriers such as limited sensitivity, selectivity, and multifunctionality by tailoring material properties, including tunable emission, enhanced quantum yield, and robust environmental stability. Examples of AI‐driven advancements include multilayer photonic structures, PCs with ultra‐high Q factors, and smart textiles embedded with polymer optical fiber (POF) sensors for real‐time health monitoring. These approaches promise to bridge the gap between diagnostics and therapy, enhancing wearable device performance for advanced remote health monitoring and personalized treatment.

Thin, flexible, and stretchable form factors derived from photonic NMs can further enhance the usability and comfort of wearable health devices. The usability and comfort of wearable healthcare devices during prolonged use are crucial for their successful integration into daily life and widespread adoption. Future advancements in this field must prioritize the development of ultra‐thin, flexible, and stretchable wearable devices that enhance functionality without compromising user usability and comfort. These features are vital to ensuring that wearable devices remain practical and appealing for long‐term use in various applications. On the other hand, there is a lack of research specifically addressing the usability and comfort of such devices for prolonged use, particularly for skin and eye applications. Key challenges include improving adhesion for stable contact with dynamic biological surfaces, enhancing biocompatibility to prevent irritation during extended use, and ensuring durability to withstand repeated use and environmental exposure. Reducing the size and thickness of these devices is equally important to minimize sensory interference and maximize comfort. Thus, the integration of photonic NMs will be pivotal in the successful development and widespread adoption of next‐generation wearable health technologies.

Several strategies have been proposed to enhance the usability and comfort of wearable healthcare devices during prolonged use. The use of ultra‐thin, flexible, and stretchable materials (e.g., PDMS, PU, textile, and paper) and nanoscale films (e.g., MoS_2_ and graphene) is emphasized to conform to the body's contours and reduce mechanical stiffness for improved comfort.^[^ ^194^ ^]^ Adhesion mechanisms are highlighted, stressing the need for materials that adhere securely to the skin without irritation, allow for repeated attachment, and conform to dynamic movements.^[^ ^194^, ^195^ ^]^ Breathability and thermal management are achieved with thermally conductive fillers consisting of boron nitride nanoparticles, hydrophobic coatings for sweat resistance, and breathable substrates such as paper/MXene composites and hydrogels, ensuring enhanced comfort and minimized irritation.^[^ ^194a,c^ ^]^ The importance of biocompatibility, lightweight designs, and compact device size is also emphasized to ensure long‐term wearability, particularly in sensitive applications including skin patches and smart contact lenses.^[^ ^194^, ^195^ ^]^ These strategies highlight the importance of combining photonic NMs with human‐centered design to address adhesion, biocompatibility, durability, and size/thickness, paving the way for next‐generation wearable devices that are both functional and comfortable for long‐term wear.

The systematic integration of photonic NMs into wearable devices is essential for their successful commercialization and widespread adoption. Photonic NMs enable exceptional precision in sensing and therapeutic applications, making it possible for wearable devices to perform advanced functions such as biomarker detection and real‐time fluorescence diagnostics. For instance, PC nanocavities amplify optical signals, improving biomarker sensitivity, while quantum dots offer precise diagnostics for health monitoring applications. Integration across wearable modalities, including skin‐mounted and eye‐based devices, facilitates multimodal health monitoring, offering comprehensive insights into a user's physiological and visual biomarkers. These systems have the potential to evolve into transformative closed‐loop applications, where diagnostics are seamlessly linked to therapeutic interventions. For example, a skin‐based glucose sensor could trigger an insulin delivery patch or an eye‐based sensor monitoring IOP could activate a drug‐releasing contact lens. Such closed‐loop systems rely on the unique optical properties of photonic NMs to enable seamless diagnostic and therapeutic integration.

However, commercializing wearable devices that incorporate photonic NMs requires addressing key challenges, such as ensuring long‐term durability, biocompatibility, and consistent performance. For skin‐based applications, durability against repeated bending and stretching can be enhanced through hierarchical designs, polymer composites, and AI‐optimized material configurations. For eye‐based applications, challenges such as transparency, precision, and long‐term wearability can be addressed by leveraging AI to identify optimal surface modifications, hydrogel coatings, and antibacterial treatments. Additionally, advancements in AI‐assisted design are crucial for market adoption. AI can refine material properties, optimize self‐assembly processes, and predict interactions between photonic NMs and biological systems. These tools will ensure that wearable devices meet stringent reliability and safety requirements, making them suitable for long‐term use in health applications.

For commercialization, regulatory and environmental impacts must also be considered. The regulatory landscapes and potential hurdles for the next generation of wearable devices utilizing photonic NMs can be benchmarked against the current regulatory guidelines in the USA and EU. In the US, wearable devices are regulated by the Food and Drug Administration (FDA). Determining whether a wearable qualifies as a medical device is the first step, as general wellness devices often bypass FDA regulation, while diagnostic or therapeutic wearables undergo scrutiny. Devices with machine learning capabilities face additional challenges regarding algorithm transparency and adaptability. Clinical trials, often conducted under Good Clinical Practice (GCP) standards, are resource‐intensive. The FDA also mandates software validation under Software as a Medical Device (SaMD) guidelines to ensure the reliability and security of wearable technologies. Post‐market requirements include adherence to surveillance obligations, with adverse events tracked via the Manufacturer and User Facility Device Experience (MAUDE) database. Data privacy and security are critical, requiring compliance with the Health Insurance Portability and Accountability Act (HIPAA) for protected health information (PHI) and rigorous cybersecurity standards, particularly for devices relying on network or cloud connectivity.

In all 27 EU member states (excluding the UK), the Medical Device Regulation (MDR) replaced the Medical Device Directive (MDD) in May 2021, establishing stringent requirements to ensure safety, performance, and quality. The integration of software such as SaMD, especially with machine learning or advanced algorithms, further complicates compliance. Clinical evaluations must demonstrate safety, scientific validity, analytical performance, and clinical performance, which can be resource‐intensive, particularly for novel technologies. Manufacturers must also implement robust post‐market surveillance (PMS) systems and continuously report incidents and performance data. In addition, compliance with the General Data Protection Regulation (GDPR) is essential for devices handling personal health data, adding complexity to data storage, processing, and transfer. Finally, all devices must meet labeling standards and obtain CE marking to be marketed in the EU. By combining material innovation, AI‐driven design, and comprehensive system integration while addressing regulatory and environmental impacts, photonic NM‐enabled wearable devices are poised to revolutionize health. These advancements promise to improve early diagnosis, enable precise therapy, and facilitate continuous health monitoring, establishing photonic NMs as foundational components in the future of personalized and non‐invasive medical technology.

## Conflict of Interest

The authors declare no conflict of interest.
